# Hydrogenation Versus Hydrosilylation: The Substantial Impact of a Palladium Capsule on the Catalytic Outcome

**DOI:** 10.3390/molecules29204910

**Published:** 2024-10-17

**Authors:** Maxime Steinmetz, Rachel Schurhammer, Christophe Gourlaouen, David Sémeril

**Affiliations:** 1 Synthèse Organométallique et Catalyse, UMR-CNRS 7177, Institut de Chimie de Strasbourg, Strasbourg University, 67008 Strasbourg, France; 2 Laboratoire de Modélisation et Simulations Moléculaires, UMR-CNRS 7140, Chimie de la Matière Complexe, Strasbourg University, 67008 Strasbourg, France

**Keywords:** metallo-capsule, resorcin[4]arene, palladium, homogeneous catalysis, molecular dynamics study

## Abstract

A palladium capsule, made of three cavitands, namely *P*,*P*-dichlorido{5,17-bis[5-(diphenylphosphanyl)-4(24),6(10),12(16),18(22)-tetramethylenedioxy-2,8,14,20-tetrapentylresorcin[4]arenyl-17-oxymthyl]-4(24),6(10),12(16),18(22)-tetramethylenedioxy-2,8,14,20-tetrapentylresorcin[4]arene}palladium(II) (**1**), was synthetized by coordination of the corresponding diphosphinated ligand and the palladium precursor [PdCl_2_(PhCN)_2_] in 27% yield. The obtained *P*,*P*-chelate complex was fully characterized by elemental analysis, NMR and mass spectrometry. Molecular dynamics simulations carried out on the metallo-capsule showed the structure made by the three cavitands was slightly distorted over the 1 μs of the simulation. The evaluation of the palladium capsule **1** in the reaction between arylacetylenes and Et_3_SiH in undried conditions unequivocally demonstrates a drastic change in chemoselectivity, with the formation of the partially hydrogenation product rather than the hydrosilylation products observed with complexes whose active center is more accessible, for instance [PdCl_2_(PPh_3_)_2_].

## 1. Introduction

The chemistry of molecular containers is currently undergoing considerable development [1,2,3,4,5,6,7]. These objects are ideal for forming new host–guest complexes and can also be used to confine catalytic reactions, allowing precise control of catalytic reactions. Consisting of one or more concave pockets [8], for example from resorcin[4]arenes [9,10,11], these molecules share similarities with enzymes [12,13,14]. In this context, molecular capsules that can host a metal center are the preferred choice. Their cavity mimics the internal pocket of an enzyme, allowing reactive species to be stabilized [15] and substrates to be accommodated, thereby modifying the chemo-, regio- and stereoselectivity of the catalytic reaction [16,17,18].

**Figure 1:**
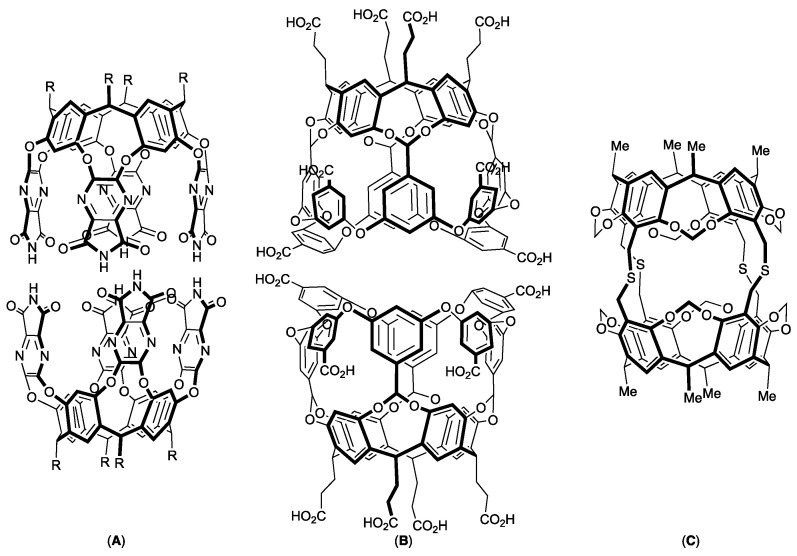
Examples of capsules (**A**–**C**) built on the resorcin[4]arene platform.

Today, there are two distinct approaches in this field. The first approach focuses on catalysts trapped in supramolecular assemblies [19,20,21,22,23,24,25,26]. Examples include dimeric capsules based on glycoluril **A** [27] or resorcin[4]arene **B** [28] (Figure 1), which form during the catalytic process. The main advantage of this approach is its simplicity. These capsules can be easily obtained from simple precursors. Capsular edifices resulting from self-assembly are the seat of weak interactions that facilitate the reversible opening of the capsules formed, thus promoting the migration of molecules from inside the capsule to the outside (and vice versa). While self-assembled entities are most often labile, making catalyst recovery difficult, recent publications have convincingly demonstrated that spatial constraints within a self-assembled cavity can result in highly selective products, including stereoselective ones [29,30,31,32,33,34]. The supramolecular approach is the best way to discover and evaluate new models of “encompassing” catalysts, without the need to synthesize covalently constructed capsule units. The logical alternative approach is to use covalent capsules, such as capsule **C** described by Cram [35]. These require more effort to synthesize, but have the advantage of greater stability and easier recovery. Examples of covalent capsules are rarer [36], especially those built on the resorcin[4]arene platform [37].

**Figure 2:**
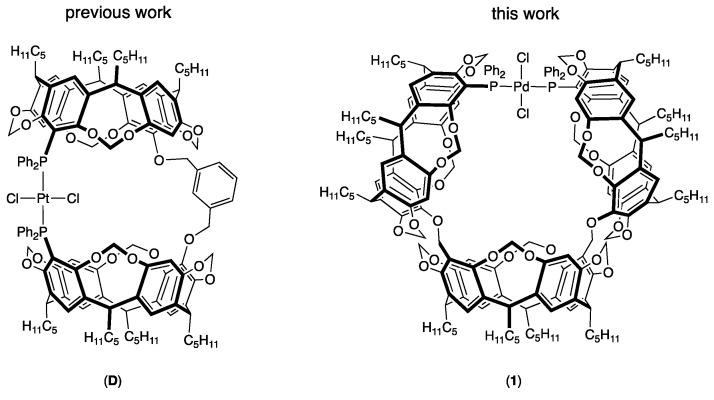
Covalent metallo-capsules **D** and **1** based on resorcin[4]arene moieties.

Our approach was based on the use of a diphosphine comprising two resorcin[4]arene cavities covalently linked by a spacer. We synthesized it with the expectation that after chelation of a transition metal by the two phosphorus atoms, the two hemispherical resorcinarenyl units could be brought together to create a capsular complex. The platinum capsular complex **D** (Figure 2) comprised, at the time of its publication, the first covalent metallo-capsules based on resorcin[4]arene moieties to function as a catalyst [37].

We tested the metallo-capsule **D** in the hydroformylation of styrene. The active species was generated in situ with the help of SnCl_2_ as a co-catalyst. Under optimized conditions, 24 h at 100 °C under 45 bar of CO/H_2_, the platinum capsule catalyzed aldehyde formation with 55% conversion and 64% regioselectivity towards the branched product. By comparison, when the reaction was carried out using the *trans*-[PtCl_2_(PPh_3_)_2_] complex, a conversion of 40% and a linear/branched ratio of 53:47 were observed. The higher activity observed with the capsular complex was undoubtedly due to the formation of intermediates whose metal center, located inside the capsule, adopted a distorted trigonal bipyramidal structure. This distortion facilitates hydride transfer to the coordinated olefin (proximity effect). The distorted trigonal bipyramidal structure is a direct result of the large P-Pt-P angle imposed by the ligand. Furthermore, steric interactions between the inner walls of the cavity and the catalytic center are slightly greater in the case of the linear Pt-alkyl intermediate. This is probably the reason for the regioselectivity observed in favor of the branched aldehyde [37].

We herein describe the synthesis and a catalytic application of the palladium capsule **1** in which the spacer between the two phosphinated resorcin[4]arene units is a cavitand (Figure 2).

## 2. Results and Discussion

### 2.1. Synthesis of the Palladium Capsule ***1***

**Scheme 1:**
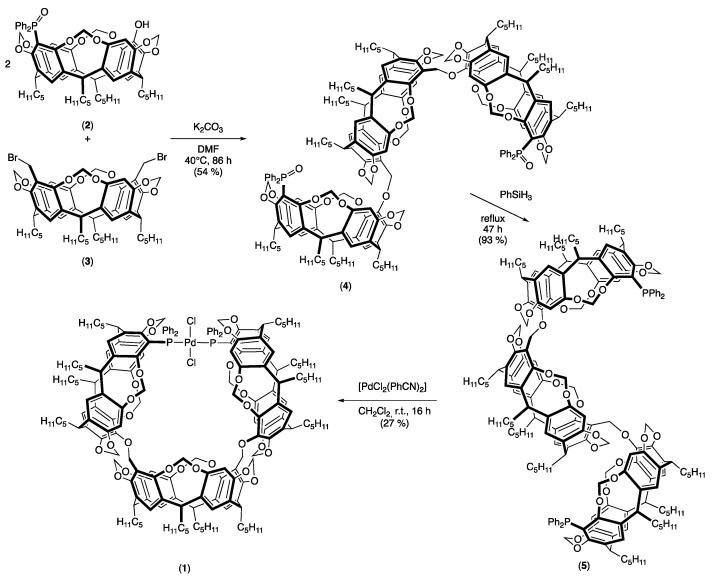
Synthesis of the palladium capsule **1**.

The palladium capsule **1** was generated in two steps from the 5-(diphenylphosphanoyl)-17-hydroxy-4(24),6(10),12(16),18(22)-tetramethylenedioxy-2,8,14,20-tetrapentylresorcin[4]arene (**2**) [37] and the 5,17-bis(bromomethyl)-4(24),6(10), 12(16),18(22)-tetramethylenedioxy-2,8,14,20-tetrapentylresorcin[4]arene (**3**) [38] (Scheme 1). The alkylation of **2** with **3** in the presence of K_2_CO_3_ in DMF led to the formation of the tris-macrocyclic compound **4** in 54% yield after 86 h at 40 °C. The bisphosphine oxide **4** was reduced to the corresponding bisphosphine **5** with phenylsilane under reflux for 47 h. After treatment with methanol, the pure bisphosphane **5** was isolated in 93% yield. In its ^31^P NMR spectrum, the PPh_2_ groups appeared as a singlet at −16.3 ppm.

**Figure 3:**
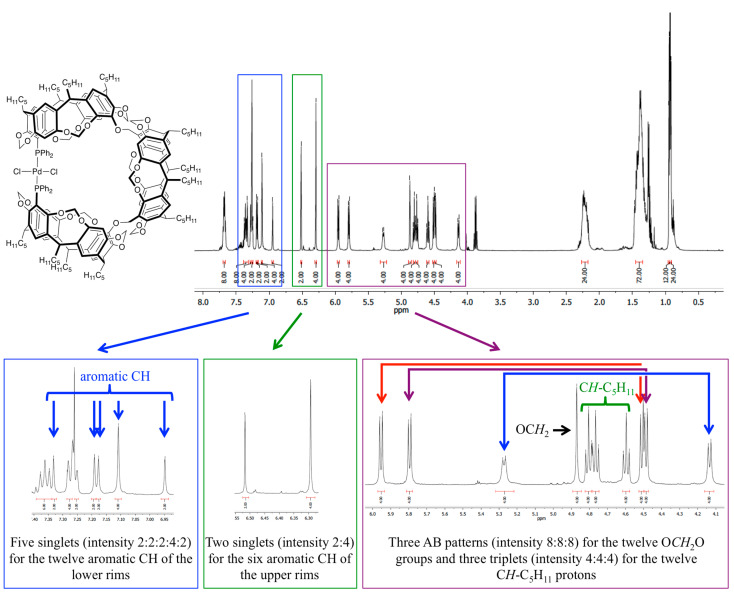
^1^H NMR spectrum of the palladium capsule **1**.

Having in hand the bisphosphine **5**, the formation of the *P*,*P*-chelate complex **1** was achieved under high-dilution conditions. Stoichiometric solutions of bisphosphine **5** and [PdCl_2_(MeCN)_2_] in CH_2_Cl_2_ were simultaneously added to a three-neck round-bottom flask containing pure CH_2_Cl_2_ over a period of three hours. In such high-dilution conditions, the palladium capsule **1** was isolated with a 27% yield. Its ^31^P NMR spectrum displayed a unique singlet at 11.6 ppm, which was consistent with a linear P-Pd-P arrangement. This was deduced from the variation in the ^31^P NMR signals of the phosphorus atoms before (in **5**) and after (in **1**) coordination (Δ*δ* = 27.9 ppm) [39]. The ^1^H and ^13^C NMR spectra were consistent with a *C*s-symmetrical structure. This undoubtedly implies rapid oscillations on the NMR time scale (*vide infra*). The metallo-capsule has 210 hydrogen atoms, yet its ^1^H NMR spectrum is relatively simple (Figure 3). The symmetry of the molecule allows us to observe only three AB patterns and three triplets, with each signal having intensities of eight and four protons, respectively, for the twelve OC*H*_2_O and C*H*(C_6_H_11_) moieties, respectively. Similarly, there are only two and six visible singlets for the six and twelve aromatic protons of the upper and lower rims, respectively, of the three cavitands.

Furthermore, the formation of the *P*,*P*-chelate complex **1** is unambiguously demonstrated with its ESI-TOF MS spectrum, which displays a strong peak at *m*/*z* = 3055.36 and 3014.33, corresponding to [M − Cl + CH_3_CN]^+^ and [M − Cl]^+^ mono-cations, respectively, and at *m*/*z* = 1526.65 and 1518.67, corresponding to [M − Cl + K]^2+^ and [M − Cl + Na]^2+^ bis-cations, respectively, with the expected and isotopic profiles.

### 2.2. Molecular Dynamics Study

**Figure 4:**
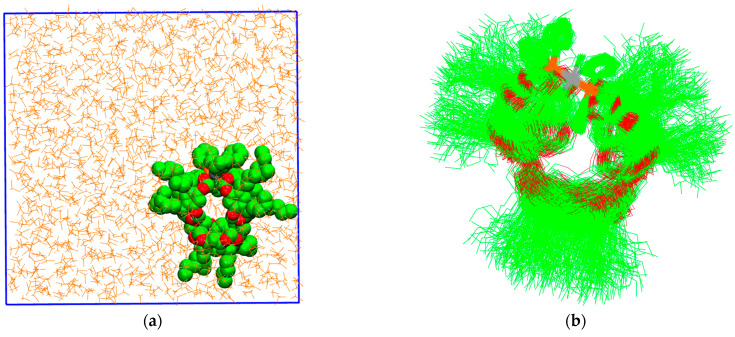
(**a**) View of the metallo-capsule **1** in the box; (**b**) Cumulative view of capsule positions during the last 250 ns, one view per ns. The root-mean-square deviations (RMSD) are 1.5 and 1.6 Å for the oxygen and carbon atoms of the resorcinarenyl skeletons, respectively.

To gain further insight into the conformational stability of the metallo-capsule **1**, molecular dynamics simulations were performed. For this purpose, the palladium complex **1** was solvated in a 59.6 × 59.6 × 59.6 Å box containing 1500 molecules of dichloromethane. The system was then simulated over a period of 1 μs to observe its evolution at a temperature of 300 K. During this time, rapid motions of the twelve pentyl chains and slight distortions of the structure formed by the three resorcinarenyl units were observed (Figure 4).

**Figure 5:**
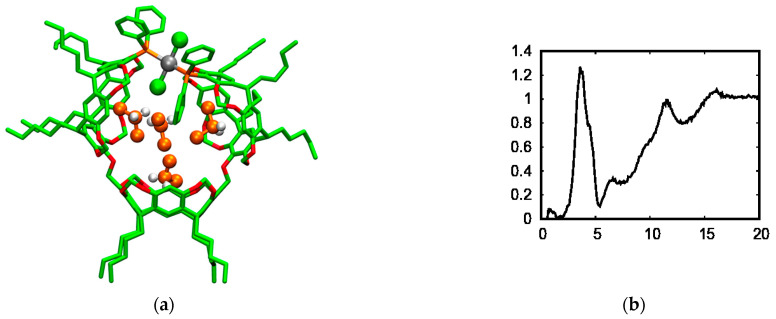
(**a**) Simulation of the metallo-capsule **1** hosted four molecules of dichloromethane; (**b**) Radial distribution of the carbon atom of the CH_2_Cl_2_ molecules around the center of mass of the capsule. The integration of the curve at 5.5 Å is 3.15 ± 0.4 molecules of CH_2_Cl_2_, which indicates that approximately 3.15 molecules of CH_2_Cl_2_ are present in the cavity during the last 100 ns of the simulation.

Furthermore, the molecular dynamic simulations demonstrated the rapid diffusion of dichloromethane molecules from the external environment into the cavity formed by the three cavitands through two portals up to 10 Å in diameter. The average number of dichloromethane molecules hosted in this cavity was 3.15 (Figure 5).

### 2.3. Catalytic Application

**Scheme 2:**
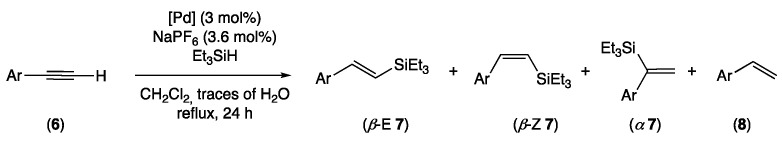
Palladium-catalyzed reaction between an arylacetylene and the triethylsilane.

The palladium-catalyzed reaction between an arylacetylene and a trialkylsilane leads generally to the hydrosilylated product in the form of a mixture of three isomers (*β*-E, *β*-Z and *α*) [40,41]. In the present work, the reaction was performed using a catalytic loading of 3 mol % in the presence of NaPF_6_ as a co-catalyst under reflux of undried CH_2_Cl_2_ for 24 h (Scheme 2).

The reaction between phenylacetylene and triethylsilane carried out with the palladium capsule **1** showed that 49% of the acetylenic substrate was consumed. The detailed ^1^H NMR analysis of the formed products clearly showed that hydrosilylated products **7** were formed in low proportions, with styrene being the main product (58% of the formed products). The *β*/*α* regioselectivity of silylated products was 1.3/1 (Table 1, entry 1). When the runs were repeated with 4-fluorophenylacetylene and 4-methoxyphenylacetylene, it was also possible to form, with lower conversions, mainly the partially hydrogenated products in proportions of 85 and 62%, respectively (Table 1, entries 2 and 3).

**Figure 6:**
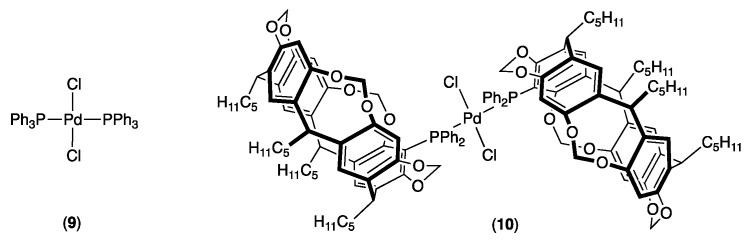
Palladium complexes **9** and **10** used to rank the catalytic activity of the metallo-capsule **1**.

The use of [PdCl_2_(PPh_3_)_2_] (**9**; Figure 6) as a catalyst resulted in a faster reaction, a full conversion of phenylacetylene and a notable, quasi-quantitative shift in chemoselectivity towards the formation of hydrosilylated compounds with a *β*/*α* ratio of 5.5 (Table 1, entry 4). Repeating the run with the palladium complex **10** (Figure 6) bearing two sterically hindered resorcinarenyl phosphines resulted in an intermediate conversion of 70% with lower chemoselectivity toward the hydrosilylated compounds (*β*/*α* ratio of 5.5) and formation of the partially hydrogenated product (28% of the formed products) (Table 1, entry 5). Adding a drop of D_2_O to the catalytic mixture reduced the conversion to 47% and drastically modified the product’s outcome. In these conditions, the partially hydrogenated compound was formed with a chemoselectivity of 39%, and the *β*/*α* ratio (5.5) of the silylated product was not affected (Table 1, entry 6). As expected, the addition of a molecular sieve reduced the amount of formed styrene to 10% and slightly increased the conversion of the catalytic reaction to 75% (Table 1, entry 7).

**Scheme 3:**
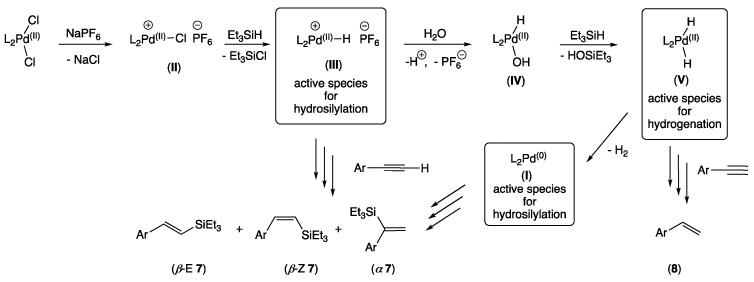
Proposed formation of active species for hydrosilylation (**I** and **III**) and partially hydrogenation (**V**).

A catalytic test with complex **10** in the absence of NaPF_6_ resulted in a lower conversion of phenylacetylene (28%; see Table S1, entry 5 in Supplementary Materials) than in the presence of the sodium salt (conversion of 70%; Table 1, entry 5). This result highlights the beneficial role of NaPF_6_ on the reactivity of complex **10**, showing that the mechanism of hydrosilylation of arylacetylenes is more complex than the one generally proposed involving the formation of [L_2_Pd^(0)^] as active species (active species **I**) [41], which is generated by reduction of [L_2_Pd^(II)^Cl_2_] with Et_3_SiH [44] (Scheme 3).

In our case, the reaction of [L_2_Pd^(II)^Cl_2_] with NaPF_6_ generated the [L_2_Pd^(II)^Cl]^+^ cation (**II**), which in the presence of Et_3_SiH led to the formation of the palladium hydride [L_2_Pd^(II)^H]^+^ (**III**), reported as an active species in the hydrosilylation of arylacetylenes [45]. In the presence of trace H_2_O, the unsaturated cationic species **III** can react to form intermediate **IV**, which after reaction with Et_3_SiH leads to the active species for the partially hydrogenation reaction, the [L_2_Pd^(II)^(H)_2_] (**V**) derivative [43]. Furthermore, the experiment proved that, in the absence of Et_3_SiH, acetophenone was formed as a unique product as the result of water addition on phenylacetylene (13% of the phenylacetylene was converted into acetophenone using palladium complex **10**; see Table S1, entry 4 in Supplementary Materials). This demonstrates that the formation of active species **V**, responsible for the partially hydrogenation product, requires the presence of Et_3_SiH.

The catalytic results clearly indicate that in the case of resorcinarenyl ligands, complexes **1** and **10**, a higher proportion of partially hydrogenated product **8** was formed. This is probably due to the fact that the lifetime of intermediate **III**, the active species for hydrosilylation, is long enough to react with water and form [L_2_Pd^(II)^(H)_2_] (**V**). The latter may either evolve, in the case of complex **9** bearing two PPh_3_ moieties, into [L_2_Pd^(0)^] (**I**) and catalyze the hydrosilylation reaction, or react quickly, in the case of macrocyclic ligands (complexes **1** and **10**), with arylacetylene previously hosted in the cavity of a resorcin[4]arene unit (spatial proximity) to lead to the partial hydrogenation products. With the PPh_3_ ligand (complex **9**), this supramolecular assistance does not exist, which makes the reaction with arylacetylene slower and therefore leaves the intermediate **V** enough time to eliminate H_2_ and form the active species for the hydrosilylation reaction (**I**).

Moreover, the sterically constrained environment clearly influences the regioselectivity of the hydrosilylated products. Catalytic results show that, when **1** is employed, the *β*/*α* ratio of the silylated compounds is lower and there is relatively more formation of *α* product. Given its more compact nature, *α* product is formed more readily when the catalytic reaction is conducted within the palladium capsule **1**. Such a proportion of the Markovnikov product (*α* **7**) is unusual for a palladium catalyst and is generally observed when ruthenium or cobalt complexes are employed [46].

## 3. Materials and Methods

General Procedure: All manipulations involving phosphorus derivatives were performed in Schlenk-type flasks under dry argon. Solvents were dried by conventional methods and distilled immediately before use. CDCl_3_ was passed down a 5 cm-thick alumina column and stored under nitrogen over molecular sieves (4 Å). Routine ^1^H, ^13^C{^1^H} and ^31^P{^1^H} spectra were recorded with an AC 500 MHz Bruker FT spectrometer. The ^1^H and ^13^C NMR spectra were referenced to residual protonated solvents (*δ* = 7.26 and 77.16 ppm, respectively). The ^31^P NMR spectroscopic data are given relative to external H_3_PO_4_. Chemical shifts and coupling constants are labeled in ppm and Hz, respectively. Elemental analyses were performed by the Service de Microanalyse, Institut de Chimie, Université de Strasbourg. Mass spectra were recorded on a Bruker MicroTOF spectrometer (ESI-TOF). 5-(Diphenylphosphanoyl)-17-hydroxy-4(24),6(10),12(16),18(22)-tetramethylenedioxy-2,8,14,20-tetrapentylresorcin[4]arene (**2**) [37], 5,17-bis(bromomethyl)-4(24),6(10),12(16),18(22)-tetramethylenedioxy-2,8,14,20-tetrapentylresorcin[4]arene (**3**) [38] and *trans*-*P,P*-dichlorido-bis[5-diphenylphosphanyl-4(24),6(10),12(16),18(22)-tetramethylenedioxy-2,8,14,20-tetrapentylresorcin[4]arene]palladium(II) (**10**) [47] were prepared by literature procedures.

### 3.1. Synthesis of 5,17-bis[5-(Diphenylphosphanoyl)-4(24),6(10),12(16),18(22)-tetramethylene-dioxy-2,8,14,20-tetrapentylresorcin[4]arenyl-17-oxymethyl]-4(24),6(10),12(16),18(22)-tetramethylenedioxy-2,8,14,20-tetrapentylresorcin[4]arene (***4***)

Cavitand **2** (290 mg, 0.28 mmol) and K_2_CO_3_ (155 mg, 1.12 mmol) were suspended in DMF (15 mL). After 0.5 h, the resorcinarene **3** (138 mg, 0.14 mmol) was added and the resulting mixture was heated at 40 °C for 86 h. The reaction mixture was then allowed to cool down to room temperature and water (40 mL) was added. The aqueous layer was extracted with CH_2_Cl_2_ (3 *×* 40 mL). The combined organic layers were washed with water (5 *×* 50 mL) and brine (50 mL), then dried over MgSO_4_. The solvent was then evaporated under reduced pressure and the crude product purified by column chromatography (CH_2_Cl_2_/Et_2_O, 9:1 *v*/*v*; *R*f = 0.57: CH_2_Cl_2_/Et_2_O, 9:1 *v*/*v*), yield 212 mg (54%). ^1^H NMR (500 MHz, CDCl_3_): *δ* = 7.70*–*7.86 (m, 8H, arom. CH, P(O)Ph_2_), 7.42*–*7.35 (m, 12H, arom. CH, P(O)Ph_2_), 7.38 (s, 2H, arom. CH, resorcinarene), 7.20 (s, 2H, arom. CH, resorcinarene), 7.15 (s, 2H, arom. CH, resorcinarene), 7.07 (s, 4H, arom. CH, resorcinarene), 6.87 (s, 2H, arom. CH, resorcinarene), 6.48 (s, 2H, arom. CH, resorcinarene), 6.38 (s, 4H, arom. CH, resorcinarene), 5.93 and 4.51 (AB spin system, 8H, OCH_2_O, ^2^*J*_HH_ = 7.5 Hz), 5.84 and 4.50 (AB spin system, 8H, OCH_2_O, ^2^*J*_HH_ = 7.5 Hz), 4.80 (t, 4H, C*H*CH_2_, ^3^*J*_HH_ = 8.0 Hz), 4.78 (s, 4H, OCH_2_C), 4.71 (t, 4H, C*H*CH_2_, ^3^*J*_HH_ = 8.0 Hz), 4.64 (t, 4H, C*H*CH_2_, ^3^*J*_HH_ = 8.0 Hz), 4.59 and 4.22 (AB spin system, 8H, OCH_2_O, ^2^*J*_HH_ = 7.5 Hz), 2.33*–*2.21 (m, 16H, CHC*H*_2_CH_2_), 2.17*–*2.10 (m, 8H, CHC*H_2_*CH_2_), 1.46*–*1.32 (m, 72H, C*H*_2_C*H*_2_C*H*_2_CH_3_), 0.94 (t, 12H, CH_2_C*H*_3_, ^3^*J*_HH_ = 7.0 Hz), 0.93 (t, 12H, CH_2_C*H*_3_, ^3^*J*_HH_ = 7.0 Hz), 0.90 (t, 12H, CH_2_C*H*_3_, ^3^*J*_HH_ = 7.0 Hz); ^13^C{^1^H} NMR (126 MHz, CDCl_3_): *δ* = 156.52*–*114.43 (arom. C’s), 100.29 (s, OCH_2_O), 100.12 (s, OCH_2_O), 100.02 (s, OCH_2_O), 65.94 (s, O*C*H_2_C), 36.83 (s, *C*HCH_2_), 36.81 (s, *C*HCH_2_), 36.53 (s, *C*HCH_2_), 32.25 (s, *C*H_2_CH_2_CH_3_), 32.19 (s, *C*H_2_CH_2_CH_3_), 32.17 (s, *C*H_2_CH_2_CH_3_), 30.25 (s, CH*C*H_2_), 30.21 (s, CH*C*H_2_), 30.11 (s, CH*C*H_2_), 27.78 (s, CHCH_2_*C*H_2_), 27.71 (s, CHCH_2_*C*H_2_), 22.85 (s, *C*H_2_CH_3_), 22.82 (s, *C*H_2_CH_3_), 14.28 (s, CH_2_*C*H_3_), 14.25 (s, CH_2_*C*H_3_); ^31^P{^1^H} NMR (202 MHz, CDCl_3_): *δ* = 23.1 (s, P(O)Ph_2_) ppm. Elemental analysis calculated (%) for C_182_H_210_O_28_P_2_ (2907.54): C 75.18, H 7.28; found: C 74.92, H 7.50.

### 3.2. Synthesis of 5,17-bis[5-(Diphenylphosphanyl)-4(24),6(10),12(16),18(22)-tetramethylene-dioxy-2,8,14,20-tetrapentylresorcin[4]arenyl-17-oxymethyl]-4(24),6(10),12(16),18(22)-tetramethylenedioxy-2,8,14,20-tetrapentylresorcin[4]arene (***5***)

Cavitand **4** (177 mg, 0.06 mmol) was diluted in phenylsilane (3 mL) and the resulting suspension was refluxed for 47 h. The volatiles were then evaporated under reduced pressure and the crude product was dissolved in CH_2_Cl_2_ (1 mL) and precipitated with methanol (5 mL). The white precipitate was filtered, washed with methanol (3 × 3 mL) and dried in a vacuum to produce **5** as a white solid, yield 163 mg (93%). ^1^H NMR (500 MHz, CDCl_3_): *δ* = 7.32*–*7.24 (m, 20H, arom. CH, PPh_2_), 7.20 (s, 2H, arom. CH, resorcinarene), 7.19 (s, 2H, arom. CH, resorcinarene), 7.15 (s, 2H, arom. CH, resorcinarene), 7.08 (s, 4H, arom. CH, resorcinarene), 6.91 (s, 2H, arom. CH, resorcinarene), 6.51 (s, 2H, arom. CH, resorcinarene), 6.36 (s, 4H, arom. CH, resorcinarene), 5.94 and 4.49 (AB spin system, 8H, OCH_2_O, ^2^*J*_HH_ = 7.5 Hz), 5.80 and 4.36 (AB spin system, 8H, OCH_2_O, ^2^*J*_HH_ = 7.0 Hz), 4.82 (t, 4H, C*H*CH_2_, ^3^*J*_HH_ = 8.0 Hz), 4.77 (s, 4H, OCH_2_C), 4.75 and 4.08 (AB spin system, 8H, OCH_2_O, ^2^*J*_HH_ = 7.5 Hz), 4.72 (t, 4H, C*H*CH_2_, ^3^*J*_HH_ = 8.5 Hz), 4.70 (t, 4H, C*H*CH_2_, ^3^*J*_HH_ = 8.3 Hz), 2.31*–*2.22 (m, 16H, CHC*H*_2_CH_2_), 2.19*–*2.12 (m, 8H, CHC*H*_2_CH_2_), 1.48*–*1.32 (m, 72H, C*H*_2_C*H*_2_C*H*_2_CH_3_), 0.94 (t, 12H, CH_2_C*H*_3_, ^3^*J*_HH_ = 7.5 Hz), 0.91 (t, 24H, CH_2_C*H*_3_, ^3^*J*_HH_ = 7.0 Hz); ^13^C{^1^H} NMR (126 MHz, CDCl_3_): *δ* = 157.21*–*114.96 (arom. C’s), 100.07 (s, OCH_2_O), 99.97 (s, OCH_2_O), 99.42 (s, OCH_2_O), 66.07 (s, O*C*H_2_C), 36.90 (s, *C*HCH_2_), 36.84 (s, *C*HCH_2_), 36.77 (s, *C*HCH_2_), 32.24 (s, *C*H_2_CH_2_CH_3_), 32.22 (s, *C*H_2_CH_2_CH_3_), 32.13 (s, *C*H_2_CH_2_CH_3_), 30.15 (s, CH*C*H_2_), 27.78 (s, CHCH_2_*C*H_2_), 27.77 (s, CHCH_2_*C*H_2_), 27.69 (s, CHCH_2_*C*H_2_), 22.88 (s, *C*H_2_CH_3_), 22.87 (s, *C*H_2_CH_3_), 22.85 (s, *C*H_2_CH_3_), 14.30 (s, CH_2_*C*H_3_), 14.27 (s, CH_2_*C*H_3_), 14.25 (s, CH_2_*C*H_3_); ^31^P{^1^H} NMR (202 MHz, CDCl_3_): *δ* = −16.3 (s, PPh_2_) ppm. Elemental analysis calculated (%) for C_182_H_210_O_26_P_2_ (2875.54): C 76.02, H 7.36; found: C 75.74, H 7.24.

### 3.3. Synthesis of P,P-Dichlorido{5,17-bis[5-(diphenylphosphanyl)-4(24),6(10),12(16),18(22)-tetramethylenedioxy-2,8,14,20-tetrapentylresorcin[4]arenyl-17-oxymethyl]-4(24),6(10),12(16),18(22)-tetramethylenedioxy-2,8,14,20-tetrapentylresorcin[4]arene}palladium(II) (***1***)

A solution of bisphosphine **5** (212 mg, 0.07 mmol) in CH_2_Cl_2_ (100 mL) and a solution of [PdCl_2_(MeCN)_2_] (28 mg, 0.07 mmol) in CH_2_Cl_2_ (100 mL) were added simultaneously over a period of 3 h in CH_2_Cl_2_ (1300 mL) and the resulting solution was stirred for an additional period of 16 h at room temperature. The solvent was then evaporated under reduced pressure and the crude product was purified by column chromatography (CH_2_Cl_2_; Rf = 0.87: CH_2_Cl_2_). The resulting solid was dissolved in CH_2_Cl_2_ (0.5 mL) and precipitated with Et_2_O (25 mL), and the yellow solid was collected and dried in a vacuum, yield 61 mg (27%). ^1^H NMR (500 MHz, CDCl_3_): δ = 7.70–7.66 (m, 8H, arom. CH, PPh_2_), 7.38–7.34 (m, 6H, arom. CH, PPh_2_), 7.33 (s, 2H, arom. CH, resorcinarene), 7.29–7.25 (m, 6H, arom. CH, PPh_2_), 7.19 (s, 2H, arom. CH, resorcinarene), 7.18 (s, 2H, arom. CH, resorcinarene), 7.11 (s, 4H, arom. CH, resorcinarene), 6.95 (s, 2H, arom. CH, resorcinarene), 6.52 (s, 2H, arom. CH, resorcinarene), 6.29 (s, 4H, arom. CH, resorcinarene), 5.95 and 4.51 (AB spin system, 8H, OCH_2_O, ^2^*J*_HH_ = 7.5 Hz), 5.80 and 4.49 (AB spin system, 8H, OCH_2_O, ^2^*J*_HH_ = 7.0 Hz), 5.27 and 4.13 (AB spin system, 8H, OCH_2_O, ^2^*J*_HH_ = 8.0 Hz), 4.87 (s, 4H, OCH_2_C), 4.80 (t, 4H, C*H*CH_2_, ^3^*J*_HH_ = 8.0 Hz), 4.77 (t, 4H, C*H*CH_2_, ^3^*J*_HH_ = 8.0 Hz), 4.60 (t, 4H, C*H*CH_2_, ^3^*J*_HH_ = 8.0 Hz), 2.27-2.16 (m, 24H, CHC*H*_2_CH_2_), 1.47–1.31 (m, 72H, C*H*_2_C*H*_2_C*H*_2_CH_3_), 0.93 (t, 12H, CH_2_C*H*_3_, ^3^*J*_HH_ = 7.0 Hz), 0.93 (t, 24H, CH_2_C*H*_3_, ^3^*J*_HH_ = 7.5 Hz); ^13^C{^1^H} NMR (126 MHz, CDCl_3_): *δ* = 157.99–115.28 (arom. C’s), 100.63 (s, OCH_2_O), 100.54 (s, OCH_2_O), 99.72 (s, OCH_2_O), 58.88 (s, O*C*H_2_C), 36.92 (s, *C*HCH_2_), 36.86 (s, *C*HCH_2_), 36.65 (s, *C*HCH_2_), 32.20 (s, *C*H_2_CH_2_CH_3_), 32.19 (s, *C*H_2_CH_2_CH_3_), 31.98 (s, *C*H_2_CH_2_CH_3_), (s, CH*C*H_2_), 30.48 (s, CH*C*H_2_), 30.22 (s, CH*C*H_2_), 30.14 (s, CH*C*H_2_), 27.74 (s, CHCH_2_*C*H_2_), 27.72 (s, CHCH_2_*C*H_2_), 27.56 (s, CHCH_2_*C*H_2_), 22.88 (s, *C*H_2_CH_3_), 22.85 (s, *C*H_2_CH_3_), 14.28 (s, CH_2_*C*H_3_), 14.27 (s, CH_2_*C*H_3_); ^31^P{^1^H} NMR (202 MHz, CDCl_3_): *δ* = 11.6 (s, PPh_2_) ppm. MS (ESI-TOF): *m*/*z* = 3055.36 [M − Cl + CH_3_CN]^+^, 3014.33 [M − Cl]^+^, 1526.65 [M − Cl + K]^2+^, 1518.67 [M − Cl + Na]^2+^ (expected isotopic profiles). Elemental analysis calculated (%) for C_182_H_210_O_26_P_2_PdCl_2_•Et_2_O (3126.99): C 71.44, H 7.09; found: C 71.52, H 7.15.

### 3.4. Molecular Dynamics Simulations

The different systems were simulated by classic molecular dynamics (MD) using AMBER.22 GPU software [48] in which the potential energy U is empirically described by a sum of bond, angle and dihedral deformation energies and pairwise additive 1-6-12 (electrostatic + van der Waals) interactions between non-bonded atoms.

$$\begin{array}{c} U=\sum_{bonds}k_{b}{\left(r-r_{0}\right)}^{2}+\sum_{angles}k_{\theta }{\left(\theta -\theta_{0}\right)}^{2}+\sum_{dihedrals}V_{n}\left[1+cos(n\phi -\gamma )\right]\\ +\sum_{i=1}^{N-1}\sum_{j=i+1}^{N}\left[\frac{A_{ij}}{R_{ij}^{12}}-\frac{B_{ij}}{R_{ij}^{6}}-\frac{q_{i}q_{j}}{\varepsilon_{0}r_{ij}}\right] \end{array}$$

The initial structure of the capsule was constructed by “hand” and optimized by semi-empirical method. The molecule was then solvated by 1500 molecules of CH_2_Cl_2_ (box size is around 59.6 × 59.6 × 59.6 Å).

Force field parameters come from the GAFF force field [49] and atomic charges were obtained using the RESP methodology (B3LYP geometry optimization and HF single-point methods for RESP calculation and 6-31G(d,p) basis set for H,C,P,Cl and SDD for Pd) [50]. Palladium parameters were from Kurt [51]. For CH_2_Cl_2_ we used the model of Kollmann [52]. Cross terms in van der Waals interactions were constructed using the Lorentz-Berthelot rules. 1–4 van der Waals and 1–4 electrostatic interactions were scaled by a factor of 1.2 and 2, respectively. The MD simulations were performed at 300 K, starting with random velocities. All simulations were carried out using 3D Periodic boundary conditions. An atom-based cut-off of 12 Å for non-bonded interactions was applied and long-range electrostatics were calculated using the Ewald summation method in the particle mesh Ewald (PME) approximation, while a long-range correction for vdW interaction was applied [53]. The SHAKE algorithm from AMBER was used to fix any bonds involving hydrogen atoms.

The different systems were first equilibrated by 0.5 ns of dynamics in the NPT ensemble, followed by 0.5 ns of dynamics in the NVT ensemble. Finally, production runs of 1 μs in the NVT ensemble were simulated. The system temperature was controlled using a Berendsen thermostat with a time step of 1 ps. Pressure was controlled using a Berendsen barostat. A time step of 2 fs was used to integrate the equations of motion via the Verlet leapfrog algorithm. Trajectories were saved every 20 ps. Snapshots along the trajectory were taken using VMD 1.9.4 software [54]. The trajectories were analyzed using our MDS software [55]. The average number of solvent molecules in the cavity was obtained by radial density functions (RDFs) calculated between the center of mass of the capsule and the carbon atoms of the dichloromethane solvent molecules. Integration of the RDF curves (along the dynamics) at 5.5 Å (approximate radius of the cavity) gave an average number of 3.15 ± 0.4.

### 3.5. Catalytic Tests

Under argon, the palladium complex (1.5 μmol, 3.0 mol%) was dissolved in CH_2_Cl_2_ (undried, 1.0 mL). NaPF_6_ (0.30 mg, 1.8 μmol, 3.6 mol%, weighted in air), phenylacetylene (5.5 μL, 50.0 μmol) and Et_3_SiH (20 μL, 125.0 μmol) were added and the reaction mixture was refluxed for 24 h. The solution was then concentrated in a vacuum and analyzed by ^1^H NMR. The spectra were compared with the literature [42,43].

## 4. Conclusions

Here we describe the two-step synthesis of 5,17-bis[5-(diphenylphosphanyl)-4(24),6(10),12(16),18(22)-tetramethylenedioxy-2,8,14,20-tetrapentylresorcin[4]arenyl-17-oxymthyl]-4(24),6(10),12(16),18(22)-tetramethylenedioxy-2,8,14,20-tetrapentylresorcin[4] arene (**5**) starting from two synthons previously developed in our laboratory. The strategy of coordinating the precursor [PdCl_2_(PhCN)_2_] with the two phosphorus atoms of **5** will produce a backbone replenishment of the three cavitands, resulting in the metallo-capsule **1**. Under ultra-high dilution, the *P*,*P*-chelate **1** was isolated in 27% yield. In agreement with ^1^H and ^13^C NMR analysis, which revealed *C*s pseudo-symmetry, molecular dynamics simulations realized on the palladium capsule **1** showed that the skeleton of the molecule oscillated rapidly without altering its structure.

The palladium capsule proved to be a surprisingly effective catalyst in the reaction between arylacetylenes and Et_3_SiH under undried conditions, leading mainly to partially hydrogenated derivatives rather than the expected hydrosilylated products. This is in contrast to the use of [PdCl_2_(PPh_3_)_2_], which typically results in the formation of the hydrosilylated products. Moreover, the sterically constrained environment clearly influenced the regioselectivity of the hydrosilylated products. Catalytic results show that, when **1** is employed, the *β*/*α* ratio of the silylated compounds is lower and there is relatively more formation of *α* product. Given its more compact nature, *α* product is formed more readily when the catalytic reaction is conducted within the palladium capsule **1**. The *β*/*α* ratio of the silylated products is indisputably lower when the *P*,*P*-chelate **1** is employed than when analogs devoid of capsular units are used. These observations are the result of two factors: (i) the longer lifetime of the intermediate resulting from the first step of the catalytic cycle, the [L_2_PdH]^+^ intermediate, and its lower reactivity with arylacetylenes, which allows, in the presence of water, the formation of the [L_2_Pd(H)_2_] active species of the partially hydrogenated products and (ii) steric interactions between the capsule wall and the intermediately formed Pd-alkyl units result in the formation of a higher proportion of the more compact *α-*hydrosilylated product.

Further studies will exploit the potential of metallo-capsules based on phosphinated cavitands in the chemoselective formation of carbon-heteroatom bond-forming reactions.

## Figures and Tables

**Table 1:** Palladium-catalyzed hydrosilylation of phenylacetylene [a].

Entry	Palladium Complex	Acetylenic (6)	Conversion (%) [b]	Product Distribution [b]			
				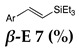	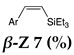	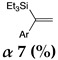	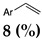
1	**1**	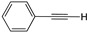	49	18	0	14	58
2	**1**	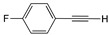	11	10	0	5	85
3	**1**	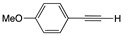	26	32	0	6	62
4	**9**	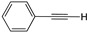	100	69	13	15	3
5	**10**	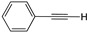	70	57	4	11	28
6 [c]	**10** + D_2_O	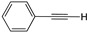	47	48	3	9	39 (D:H ratio = 83:17)
7 [d]	**10** + molecular sieve	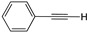	75	59	23	8	10

[a] Palladium complex (1.5 μmol, 3.0 mol%), NaPF_6_ (0.30 mg, 1.8 μmol, 3.6 mol%), arylacetylene (5.5 μL, 50.0 μmol), Et_3_SiH (20 μL, 125.0 μmol), CH_2_Cl_2_ (1.0 mL), reflux, 24 h. [b] Determinate by ^1^H NMR [42,43]. [c] Addition of D_2_O (500 μmol, 9 μL). [d] Reaction carried out in the presence of molecular sieve 4 Å.

## Data Availability

The data presented in this study are available upon request from the corresponding authors.

## Supplementary Materials

The following supporting information can be downloaded at: https://www.mdpi.com/article/10.3390/molecules29204910/s1, Characterizing data of 5,17-bis[5-(diphenylphosphanoyl)-4(24),6(10), 12(16),18(22)-tetramethylenedi-oxy-2,8,14,20-tetrapentylresorcin[4]arenyl-17-oxymethyl]-4(24), 6(10),12(16),18(22)-tetramethylenedioxy-2,8,14,20-tetrapentylresorcin[4]arene (**4**) with Figure S1: ^1^H NMR spectrum (CDCl_3_), Figure S2: ^13^C{^1^H} NMR spectrum (CDCl_3_) and Figure S3: ^31^P{^1^H} NMR spectrum (CDCl_3_); Characterizing data of 5,17-bis[5-(diphenylphosphanyl)-4(24),6(10),12(16), 18(22)-tetramethylenedi-oxy-2,8,14,20-tetrapentylresorcin[4]arenyl-17-oxymethyl]-4(24),6(10), 12(16),18(22)-tetramethylenedioxy-2,8,14,20-tetrapentylresorcin[4]arene (**5**) with Figure S4: ^1^H NMR spectrum (CDCl_3_), Figure S5: ^13^C{^1^H} NMR spectrum (CDCl_3_) and Figure S6: ^31^P{^1^H} NMR spectrum (CDCl_3_); Characterizing data of *P,P*-dichlorido{5,17-bis[5-(diphenylphosphanyl)-4(24),6(10),12(16),18(22)-tetramethylenedioxy-2,8,14,20-tetrapentylresorcin[4]arenyl-17-oxymethyl]-4(24),6(10),12(16),18(22)-tetramethylenedioxy-2,8,14,20-tetrapentylresorcin[4]arene}palladium(II) (**1**) with Figure S7: ^1^H NMR spectrum (CDCl_3_), Figure S8: ^13^C{^1^H} NMR spectrum (CDCl_3_), Figure S9: ^31^P{^1^H} NMR spectrum (CDCl_3_), Figure S10: ^1^H/^1^H COSY spectrum (CDCl_3_), Figure S11: Mass spectrum (ESI-TOF), Figure S12: Mass spectrum (ESI-TOF): exp. spectrum (top); calc. spectrum (bottom) for C_183_H_213_ClO_26_P_2_NPd ([M − Cl + CH_3_CN]^+^), Figure S13: Mass spectrum (ESI-TOF): exp. spectrum (top); calc. spectrum (bottom) for C_182_H_210_ClO_26_P_2_Pd ([M − Cl]^+^), Figure S14: Mass spectrum (ESI-TOF): exp. spectrum (top); calc. spectrum (bottom) for C_182_H_210_ClO_26_P_2_PdK ([M − Cl + K]^2+^) and Figure S15: Mass spectrum (ESI-TOF): exp. spectrum (top); calc. spectrum (bottom) for C_182_H_210_ClO_26_P_2_PdNa ([M − Cl + Na]^2+^); Catalysis with Table S1: Control experiments; Capsule structure.

## References

[1] Turner D.R., Pastor A., Alajarin M., Steed J.W., Mingos D.M.P. (2004). Molecular containers: Design approaches and applications. Supramolecular Assembly via Hydrogen Bonds I.

[2] Besset T., Gramage-Doria R., Reek J.N.H. (2013). Transition-metal encapsulation within supramolecular diphosphine capsules. Curr. Org. Chem..

[3] Deraedt C., Astruc D. (2016). Supramolecular nanoreactors for catalysis. Coord. Chem. Rev..

[4] Mouarrawis V., Plessius R., van der Vlugt J.I., Reek J.N.H. (2018). Confinement effects in catalysis using well-defined materials and cages. Front. Chem..

[5] Jongkind L.J., Caumes X., Hartendorp A.P.T., Reek J.N.H. (2018). Ligand template strategies for catalyst encapsulation. Acc. Chem. Res..

[6] Roland S., Suarez J.M., Sollogoub M. (2018). Confinement of metal-*N*-heterocyclic carbene complexes to control reactivity in catalytic reactions. Chem. Eur. J..

[7] Linnebank P.R., Poole D.A., Kluwer A.M., Reek J.N.H. (2023). A substrate descriptor based approach for the prediction and understanding of the regioselectivity in caged catalyzed hydroformylation. Faraday Discuss..

[8] Szumna A. (2010). Inherently chiral concave molecules-from synthesis to applications. Chem. Soc. Rev..

[9] Yamanaka M., Kobayashi K. (2013). Capsular assemblies of calix[4]resorcinarene-based cavitands. Asian J. Org. Chem..

[10] Harada K., Sekiya R., Haino T. (2020). A regulable internal cavity inside a resorcinarene-based hemicarcerand. Chem. Eur. J..

[11] Wang K., Liu Q., Zhou L., Sun H., Yao X., Hu X.-Y. (2023). State-of-the-art and recent progress in resorcinarene-based cavitand. Chin. Chem. Lett..

[12] Wang A.J., Clary K.N., Bergman R.G., Raymond K.N., Toste F.D. (2013). A supramolecular approach to combining enzymatic and transition metal catalysis. Nat. Chem..

[13] Bistri O., Reinaud O. (2015). Supramolecular control of transition metal complexes in water by a hydrophobic cavity: A bio-inspired strategy. Org. Biomol. Chem..

[14] Shteinman A.A. (2023). Metallocavitins as advanced enzyme mimics and promising chemical catalysts. Catalysts.

[15] Galan A., Ballester P. (2016). Stabilization of reactive species by supramolecular encapsulation. Chem. Soc. Rev..

[16] Zhang D., Jamieson K., Guy L., Gao G., Dutasta J.-P., Martinez A. (2017). Tailored oxido-vanadium(V) cage complexes for selective sulfoxidation in confined spaces. Chem. Sci..

[17] Jans A.C.H., Caumes X., Reek J.N.H. (2019). Gold catalysis in (supra)molecular cages to control reactivity and selectivity. ChemCatChem.

[18] Lorenzetto T., Bordignon F., Munarin L., Mancin F., Fabris F., Scarso A. (2023). Substrate selectivity imparted by self-assembled molecular containers and catalysts. Chem. Eur. J..

[19] Rudkevich D.M., Rebek J. (1999). Deepening cavitands. Eur. J. Org. Chem..

[20] Koblenz T.S., Dekker H.L., de Koster C.G., van Leeuwen P.W.N.M., Reek J.N.H. (2011). Bis(metallo) capsules based on two ionic diphosphines. Chem. Asian J..

[21] Schröder T., Sahu S.N., Mattay J., Albrecht M., Hahn E. (2012). Molecular capsules derived from resorcin[4]arenes by metal-coordination. Chemistry of Nanocontainers.

[22] Jiang X.-F., Cui Y.-X., Yu S.-Y. (2014). Detteersign and synthesis of a new kind of cavitand: Tetrapyrazolylcalix[4]arenes and their supramolecular assemblies. Synlett.

[23] Cholewa P.P., Dalgarno S.J. (2014). Metal-organic calixarene capsules: The evolution of controlled assembly. CrystEngComm.

[24] Mendez-Arroyo J., d’Aquino A.I., Chinen A.B., Manraj Y.D., Mirkin C.A. (2017). Reversible and selective encapsulation of dextromethorphan and *β*-estradiol using an asymmetric molecular capsule assembled via the weak-link approach. J. Am. Chem. Soc..

[25] Chen M., Wang J., Liu D., Jiang Z., Liu Q., Wu T., Liu H., Yu W., Yan J., Wang P. (2018). Highly stable spherical metallo-capsule from a branched hexapodal terpyridine and its self-assembled berry-type nanostructure. J. Am. Chem. Soc..

[26] Sekiya R., Harada K., Nitta N., Haino T. (2022). Resorcinarene-based supramolecular capsules: Supramolecular functions and applications. Synlett.

[27] Körner S.K., Tucci F.C., Rudkevich D.M., Heinz T., Rebek J. (2000). A self-assembled cylindrical capsule: New supramolecular phenomena through encapsulation. Chem. Eur. J..

[28] Gibb C.L.D., Gibb B.C. (2004). Well-defined, organic nanoenvironments in water:  the hydrophobic effect drives a capsular assembly. J. Am. Chem. Soc..

[29] Kobayashi K., Yamanaka M. (2015). Self-assembled capsules based on tetrafunctionalized calix[4]resorcinarene cavitands. Chem. Soc. Rev..

[30] Catti L., Zhang Q., Tiefenbacher K. (2016). Advantages of catalysis in self-assembled molecular capsules. Chem. Eur. J..

[31] Zhang Q., Catti L., Tiefenbacher K. (2018). Catalysis inside the hexameric resorcinarene capsule. Acc. Chem. Res..

[32] Fang Y., Powell J.A., Li E., Wang Q., Perry Z., Kirchon A., Yang X., Xiao Z., Zhu C., Zhang L. (2019). Catalytic reactions within the cavity of coordination cages. Chem. Soc. Rev..

[33] Pappalardo A., Puglisi R., Sfrazzetto G.T. (2019). Catalysis inside supramolecular capsules: Recent developments. Catalysts.

[34] Lorenzetto T., Fabris F., Scarso A. (2022). A resorcin[4]arene hexameric capsule as a supramolecular catalyst in elimination and isomerization reactions. Beilstein J. Org. Chem..

[35] Cram D.J., Karbach S., Kim Y.H., Baczynskyj K., Kallemeyn G.W. (1985). Shell closure of two cavitands forms carcerand complexes with components of the medium as permanent guests. J. Am. Chem. Soc..

[36] Yang J., Chatelet B., Hérault D., Dutasta J.-P., Martinez A. (2018). Covalent cages with inwardly directed reactive centers as confined metal and organocatalysts. Eur. J. Org. Chem..

[37] Chavagnan T., Sémeril D., Matt D., Toupet L. (2017). Cavitand chemistry*—*Towards metallocapsular catalysts. Eur. J. Org. Chem..

[38] El Moll H., Sémeril D., Matt D., Toupet L. (2010). Regioselective grafting of two -CH_2_P(X)Ph_2_ units (X = O, lone pair) onto a resorcin[4]arene-derived cavitand. Eur. J. Org. Chem..

[39] Monnereau L., Sémeril D., Matt D., Toupet L. (2010). Cavity-shaped ligands: Calix[4]arene-based monophosphanes for fast Suzuki–Miyaura cross-coupling. Chem. Eur. J..

[40] Sumida Y., Kato T., Yoshida S., Hosoya T. (2012). Palladium-catalyzed regio- and stereoselective hydrosilylation of electron-deficient alkynes. Org. Lett..

[41] Shamna S., Fairoosa J., Afsina C.M.A., Anilkumar G. (2022). Palladium-catalysed hydrosilylation of unsaturated compounds. J. Organomet. Chem..

[42] Solomonsz W.A., Rance G.A., Khlobystov A.N. (2014). Evaluating the effects of carbon nanoreactor diameter and internal structure on the pathways of the catalytic hydrosilylation reaction. Small.

[43] Duan Y., Ji G., Zhang S., Chen X., Yang Y. (2018). Additive-modulated switchable reaction pathway in the addition of alkynes with organosilanes catalyzed by supported Pd nanoparticles: Hydrosilylation versus semihydrogenation. Catal. Sci. Technol..

[44] Mirza-Aghayan M., Boukherroub R., Rahimifard M. (2008). Efficient method for the reduction of carbonyl compounds by triethylsilane catalyzed by PdCl_2_. J. Organomet. Chem..

[45] Tafazolian H., Schmidt J.A.R. (2015). Highly efficient regioselective hydrosilylation of allenes using a [(3IP)Pd(allyl)]OTf catalyst; first example of allene hydrosilylation with phenyl- and diphenylsilane. Chem. Commun..

[46] Chen J., Wei W.-T., Li Z., Lu Z. (2024). Metal-catalyzed Markovnikov-type selective hydrofunctionalization of terminal alkynes. Chem. Soc. Rev..

[47] Monnereau L., El Moll H., Sémeril D., Matt D., Toupet L. (2014). Resorcinarenyl-phosphines in Suzuki-Miyaura cross-coupling reactions of aryl chlorides. Eur. J. Inorg. Chem..

[48] Case D.A., Aktulga H.M., Belfon K., Ben-Shalom I.Y., Berryman J.T., Brozell S.R., Cerutti D.S., Cheatham T.E., Cisneros G.A., Cruzeiro V.W.D. (2022). Amber 2022.

[49] Wang J., Wolf R.M., Caldwell J.W., Kollman P.A. (2004). Development and testing of a general amber force field. J. Comput. Chem..

[50] Bayly C.I., Cieplak P., Cornell W.D., Kollman P.A. (1993). A well-behaved electrostatic potential based method using charge restraints for deriving atomic charges: The RESP model. J. Phys. Chem..

[51] Kurt B. (2023). Assign_v2: A novel bonded-force field parameterization software for square planar palladium molecular dynamics simulations. J. Biomol. Struct. Dyn..

[52] Fox T., Kollman P.A. (1998). Application of the RESP methodology in the parametrization of organic solvents. J. Phys. Chem. B.

[53] Allen M.P., Tildesley D.J. (1987). Computer Simulation of Liquids.

[54] Humphrey W., Dalke A., Schulten K. (1996). VMD: Visual molecular dynamics. J. Mol. Graph..

[55] Engler E., Wipff G., Tsoucaris G. (1996). MD-DRAW Software. Display of dynamic structures from MD simulations. Crystallography of Supramolecular Compounds.

