# Supplementary material for: Hydrogenation Versus Hydrosilylation: The Substantial Impact of a Palladium Capsule on the Catalytic Outcome
Source: Molecules. 2024 Oct 17;29(20):4910. doi: 10.3390/molecules29204910 (PMC11510042; doi:10.3390/molecules29204910)
Supplement: Supplementary file 1 [file molecules-29-04910-s001.zip › molecules-3237155-supplementary.pdf]

Hydrogenation versus hydrosilylation: the substantial impact of a pallado-capsule on the catalytic outcome

Maxime Steinmetz, Rachel Schurhammer, Christophe Gourlaouen and David Sémeril

Contents

Characterizing data of 5,17-bis[5-(diphenylphosphanoyl)-4(24),6(10),12(16),18(22)-tetramethylenedi-oxy-2,8,14,20-tetrapentylresorcin[4]arenyl-17-oxymethyl]-4(24),6(10),12(16),18(22)-tetramethylenedioxy-2,8,14,20-tetrapentylresorcin[4]arene (**4**) p 2

Figure S1. ^{1}H NMR spectrum ($CDCl_3$)

Figure S2. $^{13}C\{^{1}H\}$ NMR spectrum ($CDCl_3$)

Figure S3. $^{31}P\{^{1}H\}$ NMR spectrum ($CDCl_3$)

Characterizing data of 5,17-bis[5-(diphenylphosphanyl)-4(24),6(10),12(16),18(22)-tetramethylenedi-oxy-2,8,14,20-tetrapentylresorcin[4]arenyl-17-oxymethyl]-4(24),6(10),12(16),18(22)-tetramethylenedioxy-2,8,14,20-tetrapentylresorcin[4]arene (**5**) p 4

Figure S4. ^{1}H NMR spectrum ($CDCl_3$)

Figure S5. $^{13}C\{^{1}H\}$ NMR spectrum ($CDCl_3$)

Figure S6. $^{31}P\{^{1}H\}$ NMR spectrum ($CDCl_3$)

Characterizing data of *P,P*-dichlorido{5,17-bis[5-(diphenylphosphanyl)-4(24),6(10),12(16),18(22)- tetramethylenedioxy-2,8,14,20-tetrapentylresorcin[4]arenyl-17-oxymethyl]-4(24),6(10),12(16),18(22)-tetramethylenedioxy-2,8,14,20-tetrapentylresorcin[4]arene}palladium(II) (**1**) p 6

Figure S7. ^{1}H NMR spectrum ($CDCl_3$)

Figure S8. $^{13}C\{^{1}H\}$ NMR spectrum ($CDCl_3$)

Figure S9. $^{31}P\{^{1}H\}$ NMR spectrum ($CDCl_3$)

Figure S10. $^{1}H/^{1}H$ COSY spectrum ($CDCl_3$)

Figure S11. Mass spectrum (ESI-TOF)

Figure S12. Mass spectrum (ESI-TOF): exp. spectrum (top); calc. spectrum (bottom) for $C_{183}H_{213}ClO_{26}P_2NPd$ ($[M - Cl + CH_3CN]^+$)

Figure S13. Mass spectrum (ESI-TOF): exp. spectrum (top); calc. spectrum (bottom) for $C_{182}H_{210}ClO_{26}P_2Pd$ ($[M - Cl]^+$)

Figure S14. Mass spectrum (ESI-TOF): exp. spectrum (top); calc. spectrum (bottom) for $C_{182}H_{210}ClO_{26}P_2PdK$ ($[M - Cl + K]^{2+}$)

Figure S15. Mass spectrum (ESI-TOF): exp. spectrum (top); calc. spectrum (bottom) for $C_{182}H_{210}ClO_{26}P_2PdNa$ ($[M - Cl + Na]^{2+}$)

Catalysis p 10

Table S1. Control experiments

Capsule structure p 11

5,17-Bis[5-(diphenylphosphanoyl)-4(24),6(10),12(16),18(22)-tetramethylenedi-oxy-2,8,14,20-tetrapentylresorcin[4]arenyl-17-oxymethyl]-4(24),6(10),12(16),18(22)-tetramethylenedioxy-2,8,14,20-tetrapentylresorcin[4]arene (4)

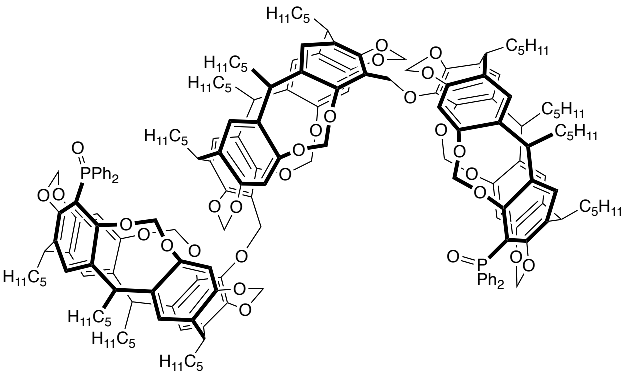

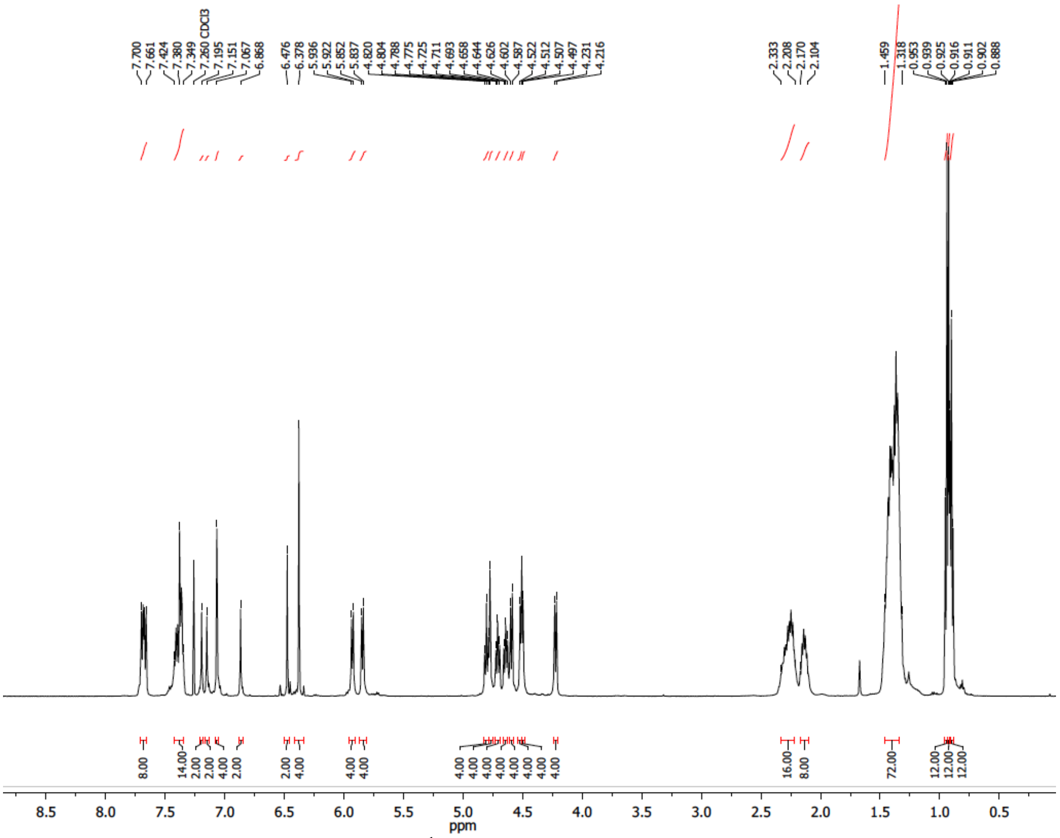

Figure S1. ^{1}H NMR spectrum ($CDCl_3$)

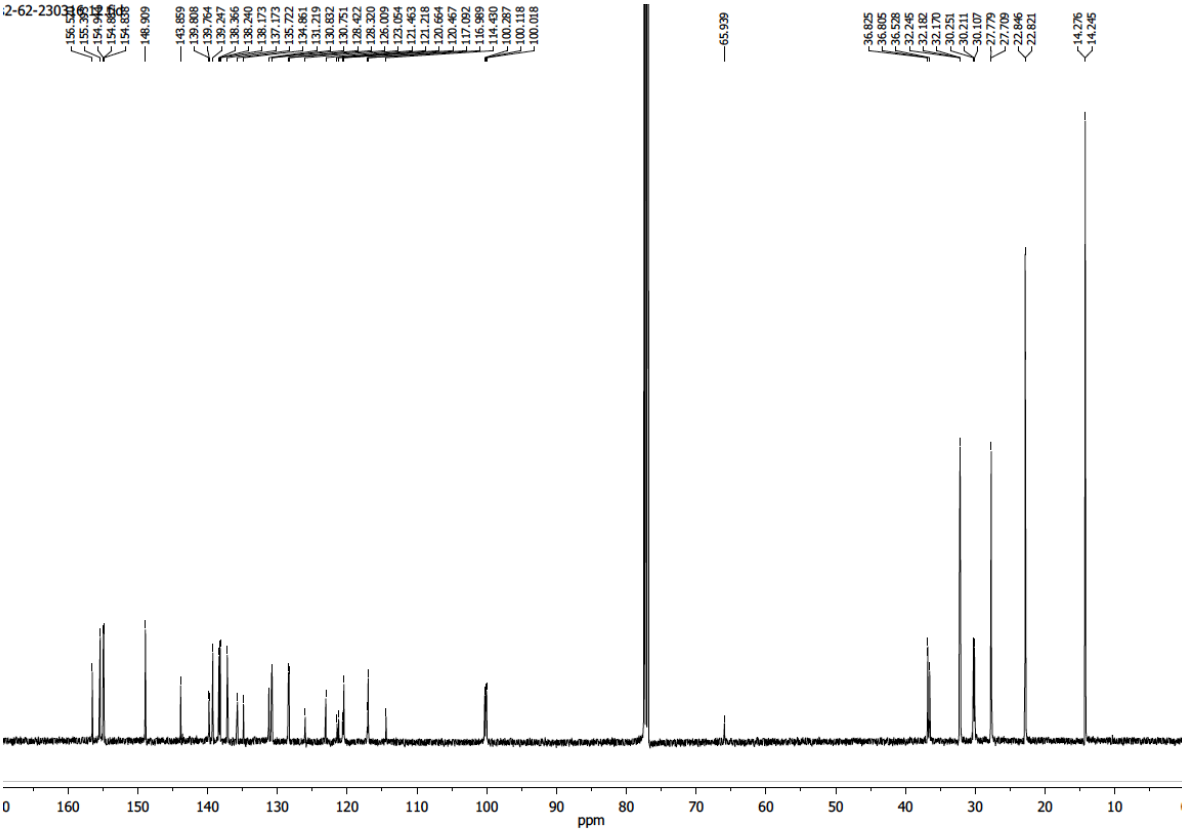

Figure S2. $^{13}C\{^{1}H\}$ NMR spectrum ($CDCl_3$)

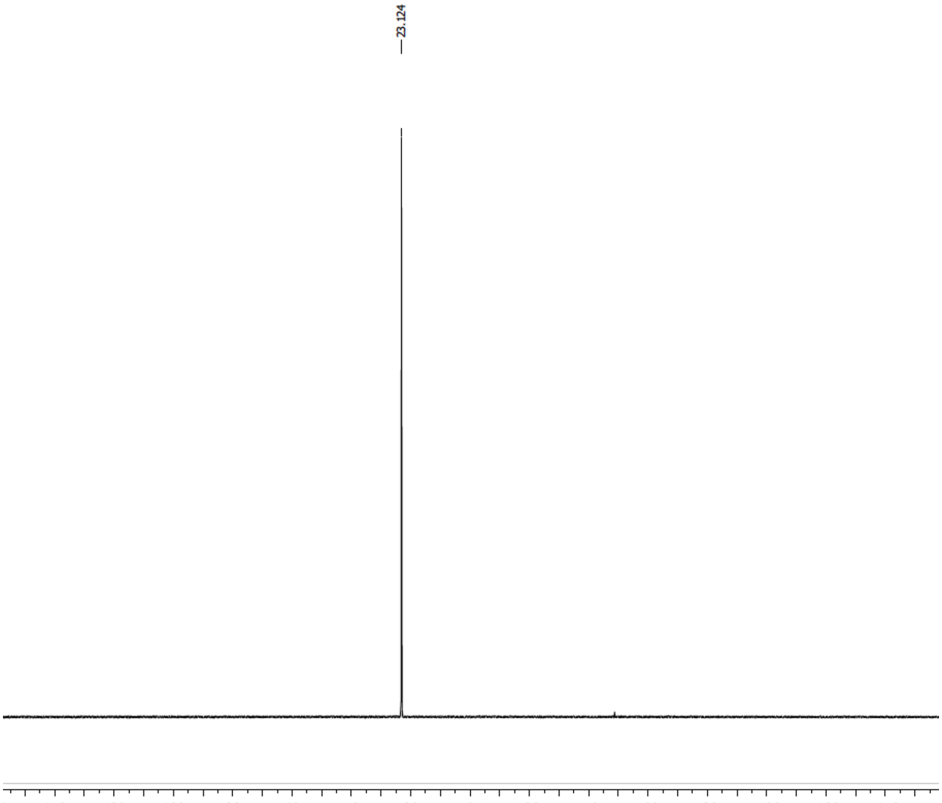

Figure S3. $^{31}P\{^{1}H\}$ NMR spectrum ($CDCl_3$)

5,17-Bis[5-(diphenylphosphanyl)-4(24),6(10),12(16),18(22)-tetramethylenedi-oxy-2,8,14,20-tetrapentylresorcin[4]arenyl-17-oxymethyl]-4(24),6(10),12(16),18(22)-tetramethylenedioxy-2,8,14,20-tetrapentylresorcin[4]arene (5)

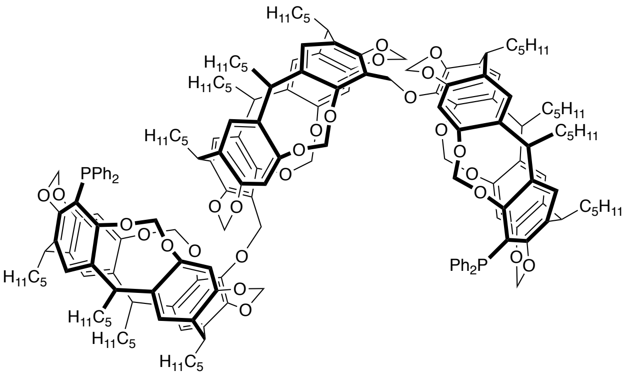

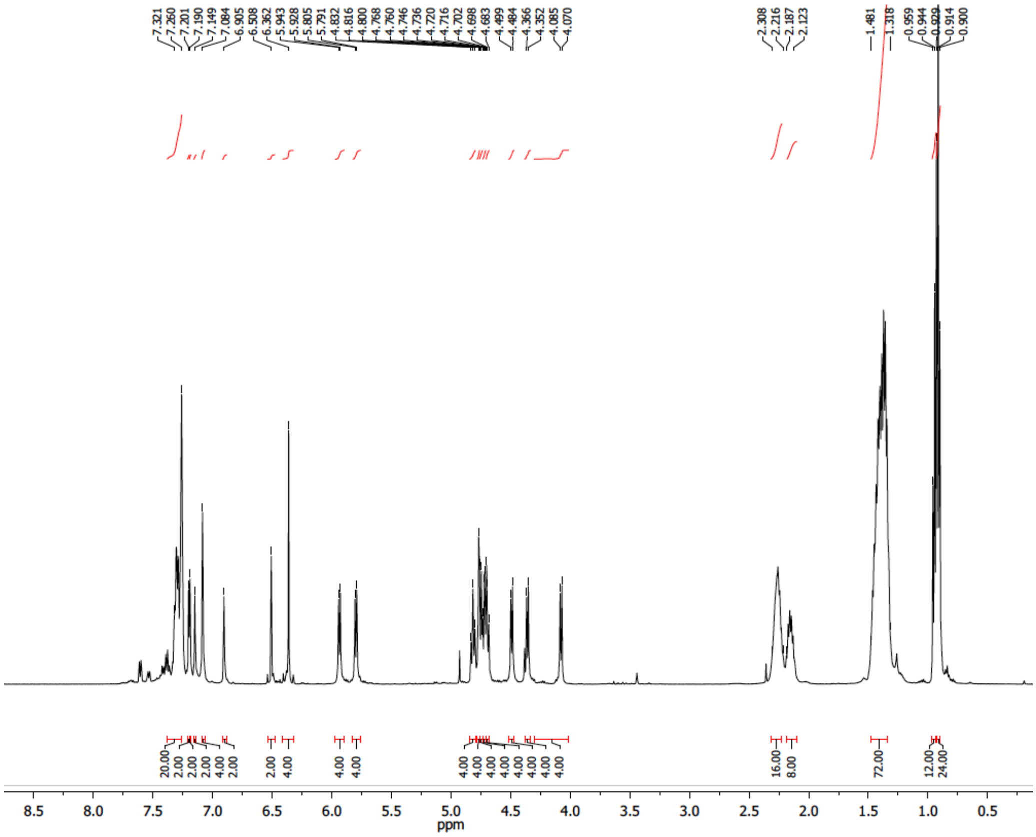

Figure S4. ^{1}H NMR spectrum ($CDCl_3$)

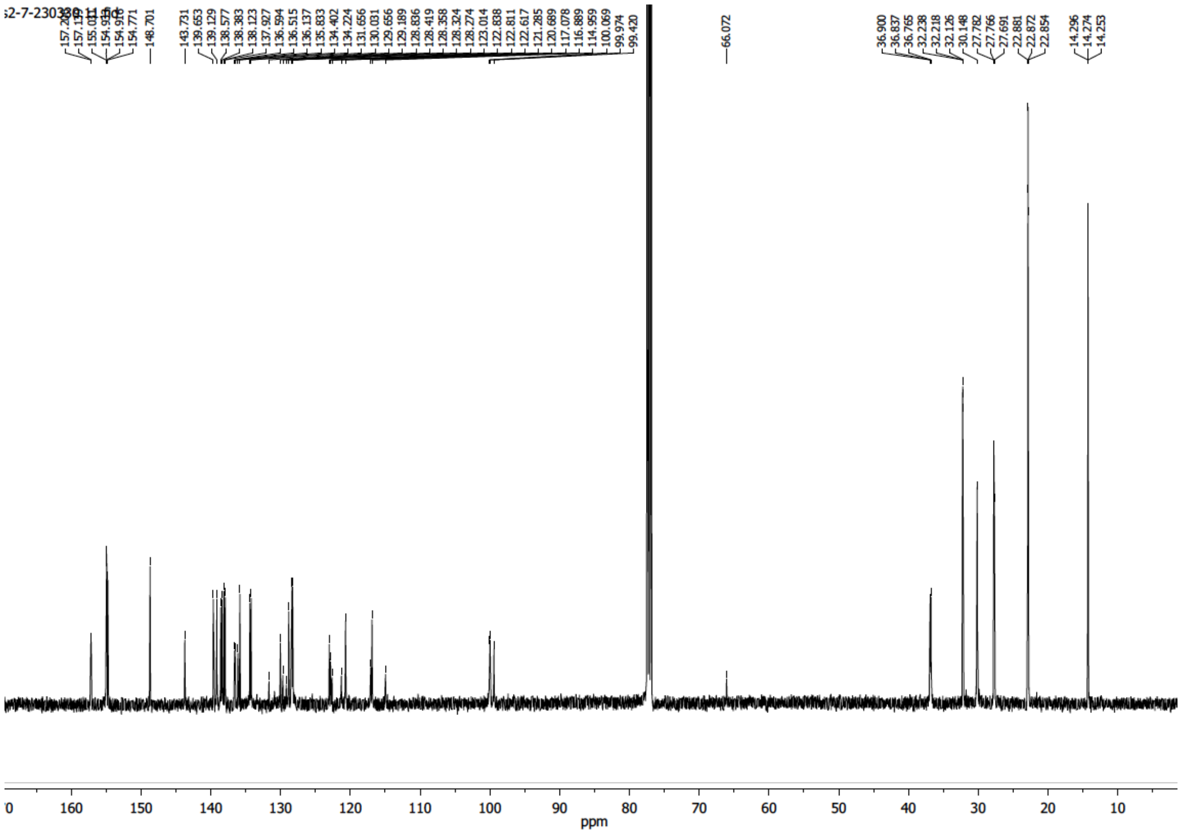

Figure S5. $^{13}C\{^1H\}$ NMR spectrum ($CDCl_3$)

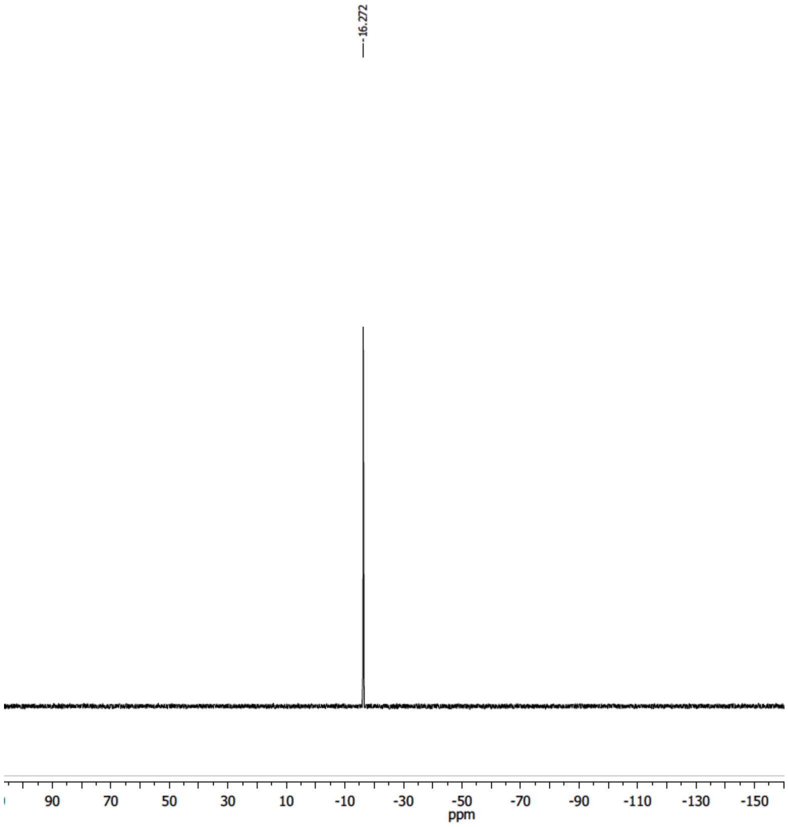

Figure S6. $^{31}P\{^1H\}$ NMR spectrum ($CDCl_3$)

_P,P_-Dichlorido{5,17-bis[5-(diphenylphosphanyl)-4(24),6(10),12(16),18(22)- tetramethylenedioxy-2,8,14,20-tetrapentylresorcin[4]arenyl-17-oxymethyl]-4(24),6(10),12(16),18(22)-tetramethylenedioxy-2,8,14,20-tetrapentylresorcin[4]arene}palladium(II) (1)

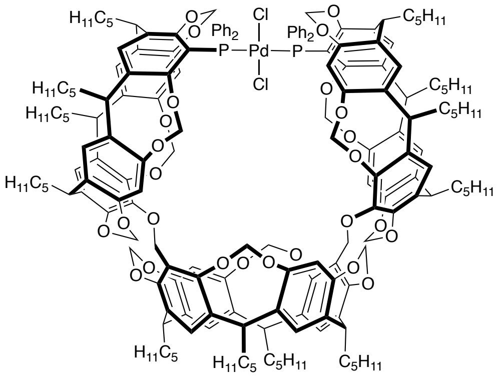

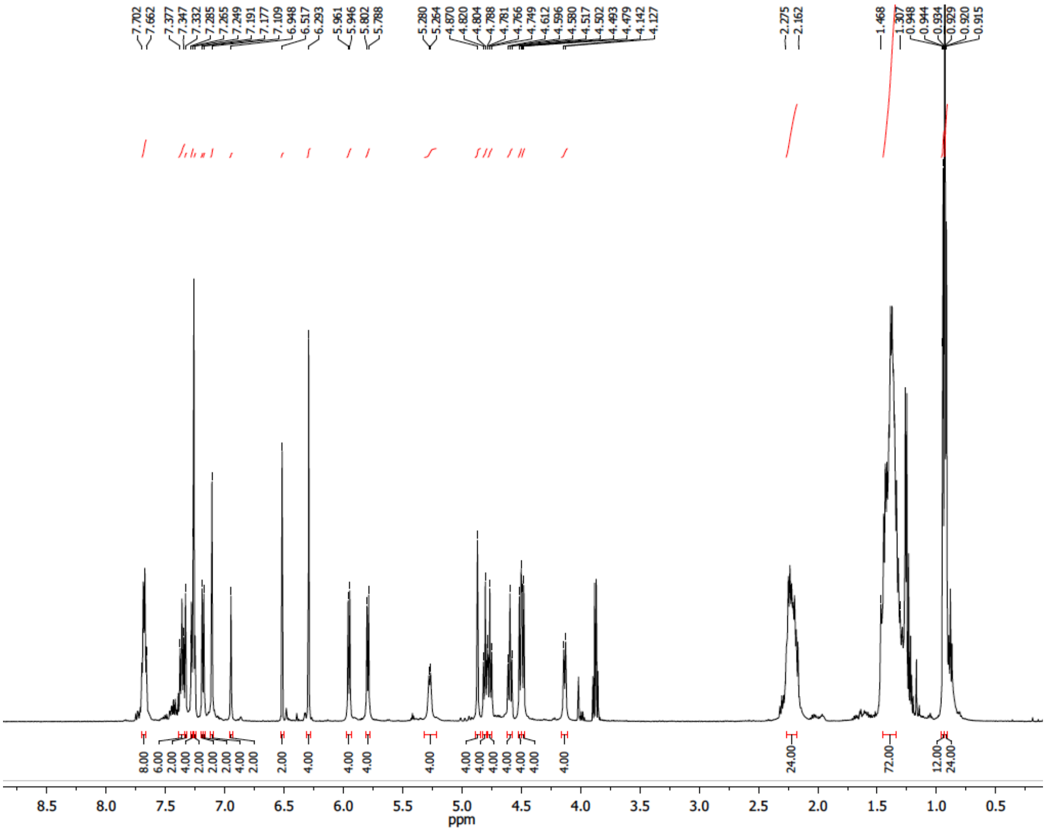

Figure S7. ${}^{1}H$ NMR spectrum ($CDCl_3$)

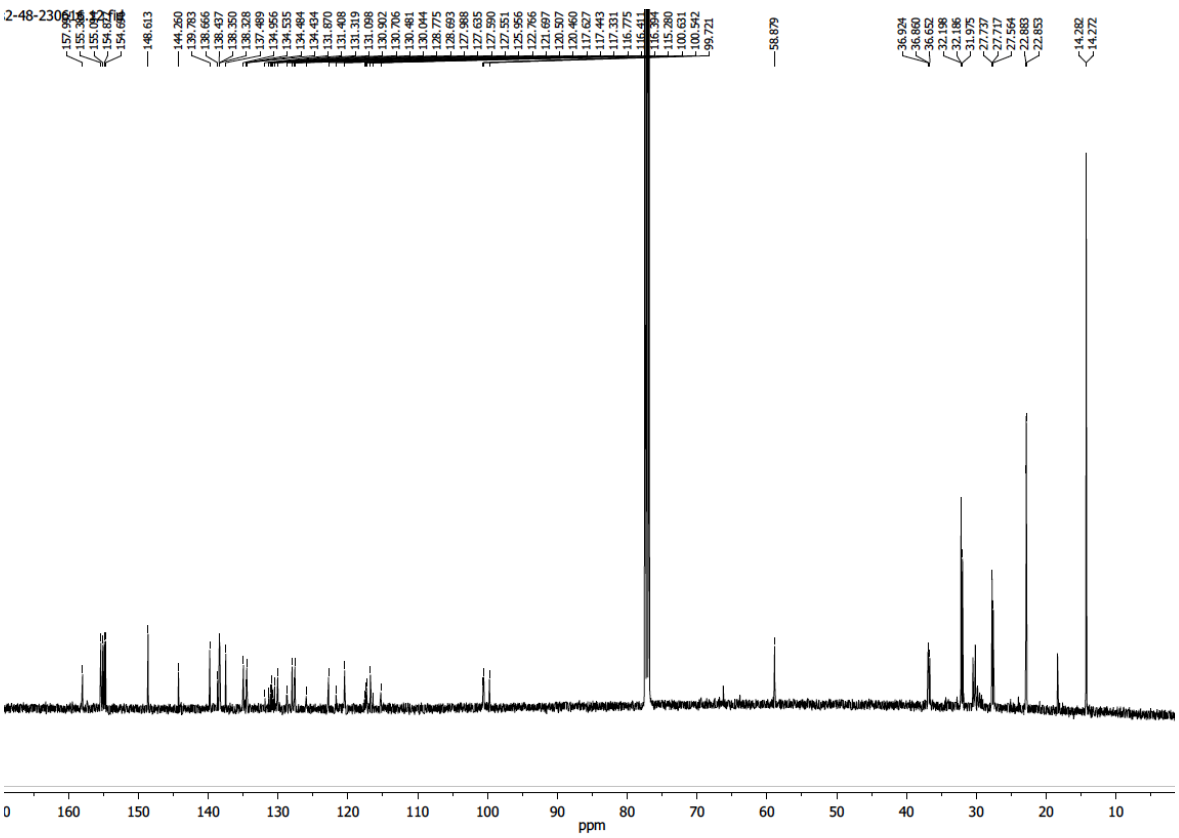

Figure S8. $^{13}C\{^1H\}$ NMR spectrum ($CDCl_3$)

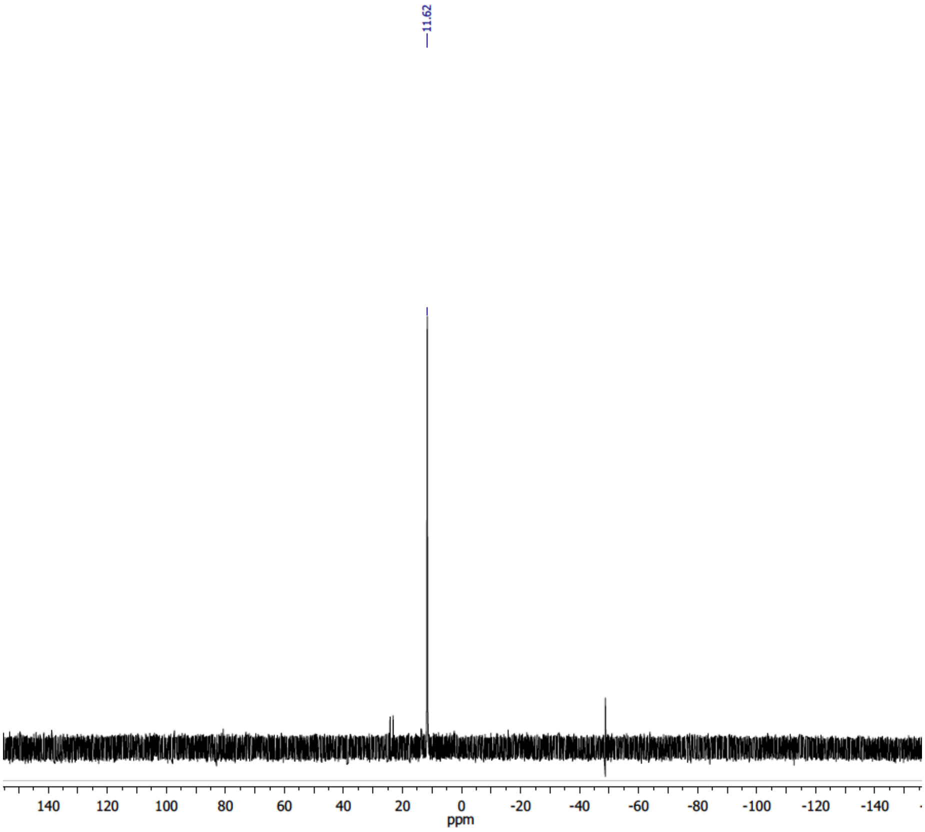

Figure S9. $^{31}P\{^1H\}$ NMR spectrum ($CDCl_3$)

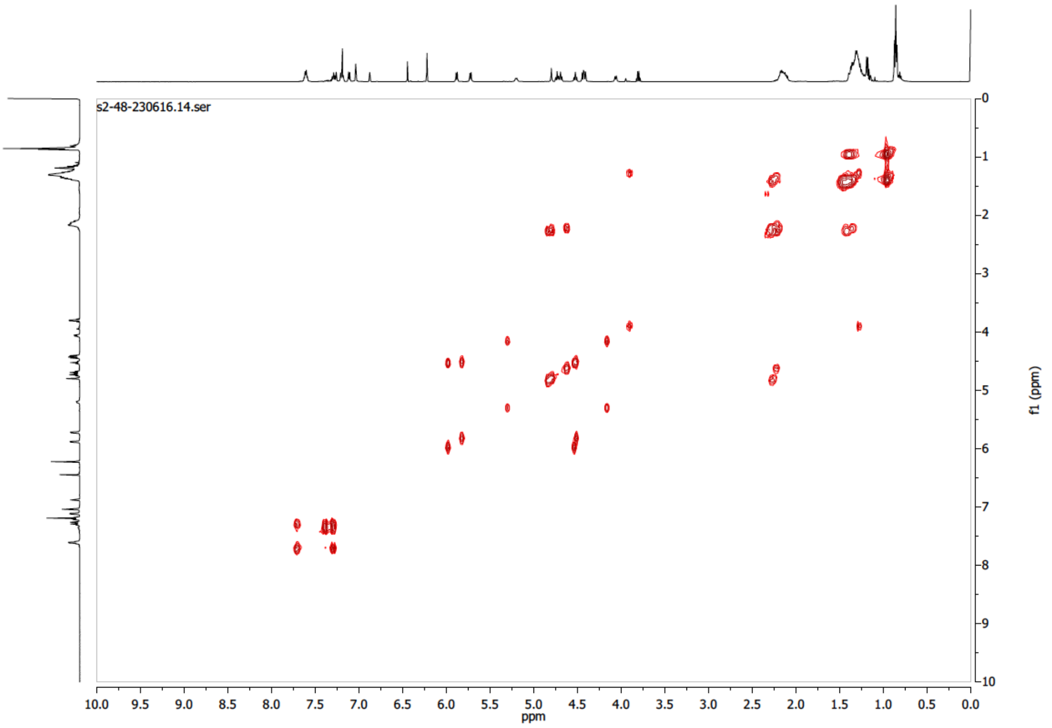

Figure S10. $^{1}H/^{1}H$ COSY spectrum ($CDCl_3$)

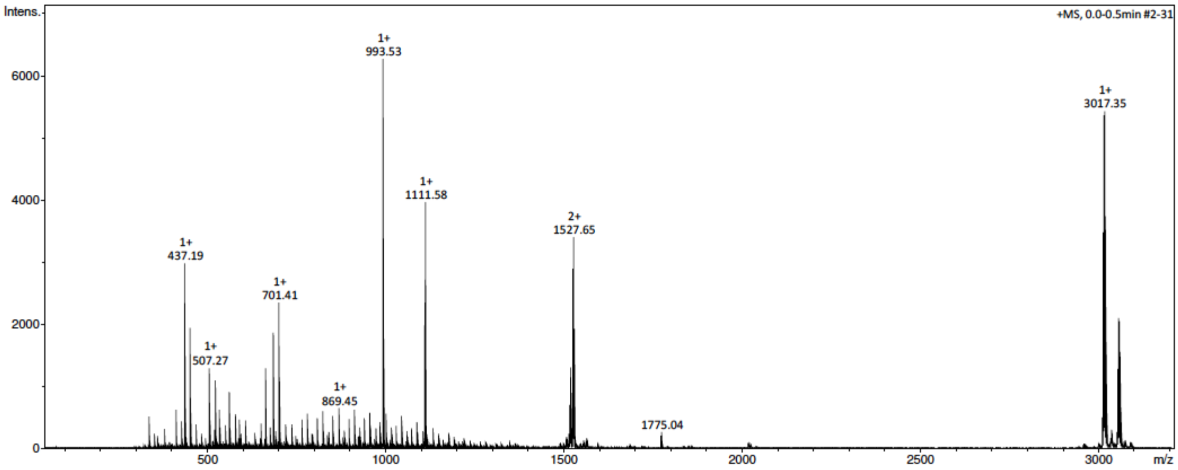

Figure S11. Mass spectrum (ESI-TOF)

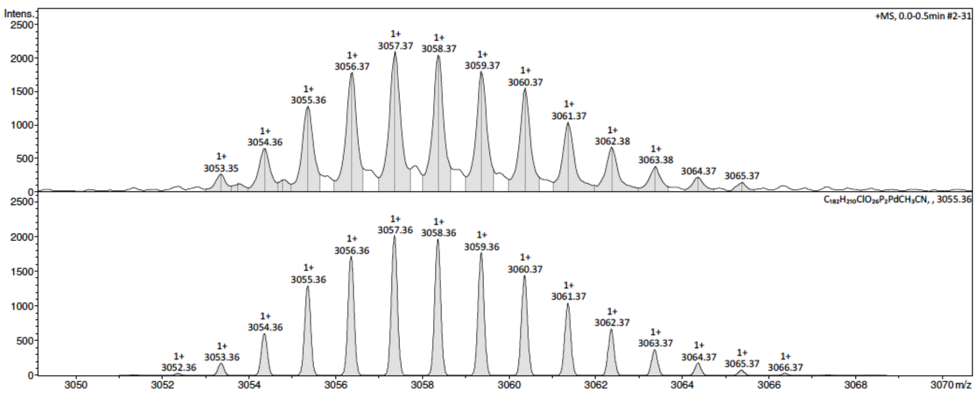

Figure S12. Mass spectrum (ESI-TOF): exp. spectrum (top);
calc. spectrum (bottom) for $C_{183}H_{213}ClO_{26}P_2NPd$ ($[M - Cl + CH_3CN]^+$)

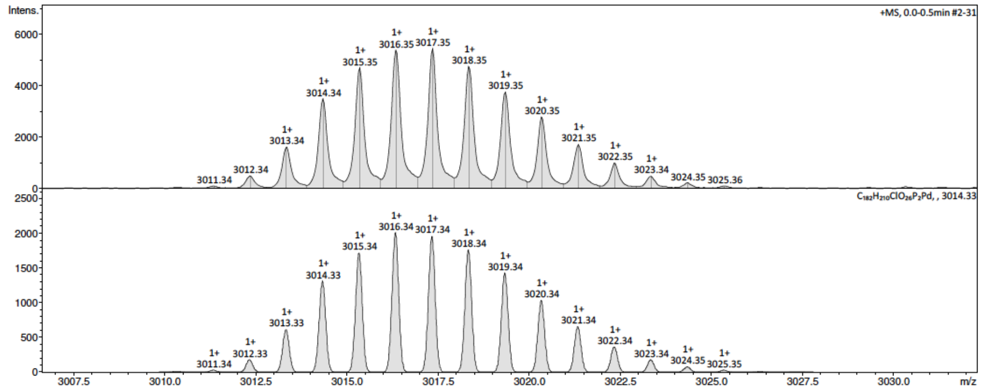

Figure S13. Mass spectrum (ESI-TOF): exp. spectrum (top); calc. spectrum (bottom) for $C_{182}H_{210}ClO_{26}P_2Pd$ ($[M - Cl]^+$)

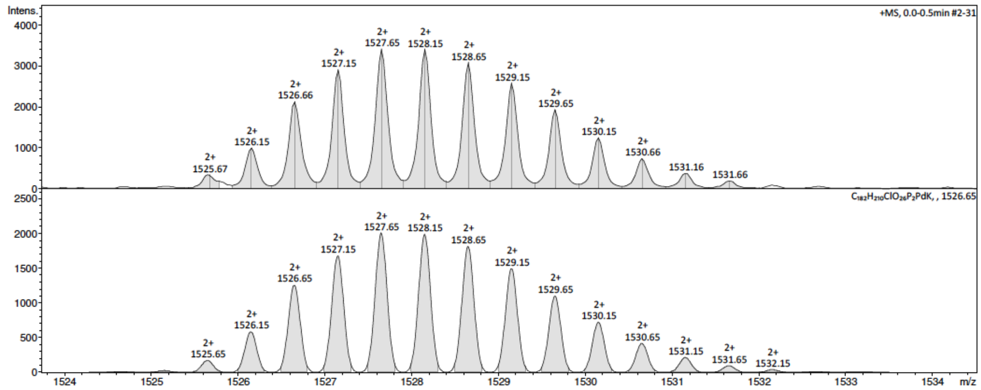

Figure S14. Mass spectrum (ESI-TOF): exp. spectrum (top); calc. spectrum (bottom) for $C_{182}H_{210}ClO_{26}P_2PdK$ ($[M - Cl + K]^{2+}$)

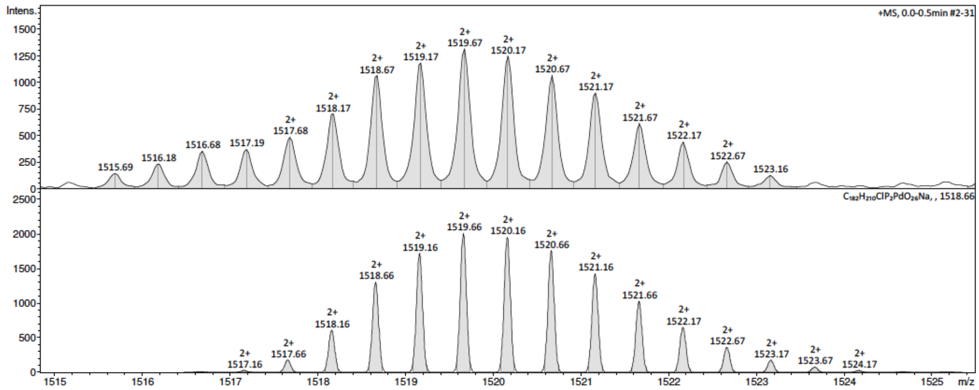

Figure S15. Mass spectrum (ESI-TOF): exp. spectrum (top); calc. spectrum (bottom) for $C_{182}H_{210}ClO_{26}P_2PdNa$ ($[M - Cl + Na]^{2+}$)

Catalysis

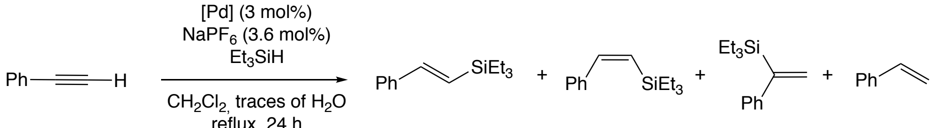

(**6**) (*β*-E **7**) (*β*-Z **7**) (*α* **7**) (**8**)

Table S1. Control experiments.

Entry	Modification of the conditions [a]	Conversion (%) [b]	**Products distribution** [b]				
			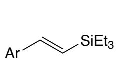 ***β*-E 7 (%)**	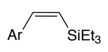 ***β*-Z 7 (%)**	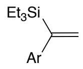 ***α* 7 (%)**	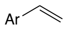 **8 (%)**	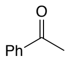 **(%)**
1	/	70	57	4	11	28	/
2	with molecular sieve	75	59	23	8	10	/
3	with D_2O (500 µmol, 9 µL)	47	48	3	9	39	/
4	without Et_3SiH	13	/	/	/	/	100
5	without $NaPF_6$	28	40	10	4	46	/

[a] Palladium complex **10** (1.5 µmol, 3.0 mol%), $NaPF_6$ (0.30 mg, 1.8 µmol, 3.6 mol%), phenylacetylene (5.5 µL, 50.0 µmol), Et_3SiH (20 µL, 125.0 µmol), CH_2Cl_2 (1.0 mL), reflux, 24 h.

[b] Determinate by ^{1}H NMR [1,2].

References

[1] Solomonsz , W.A.; Rance, G.A.; Khlobystov, A.N. Evaluating the effects of carbon nanoreactor diameter and internal structure on the pathways of the catalytic hydrosilylation reaction. *Small* **2014**, *10*, 1866-1872.

[2] Duan, Y.; Ji, G.; Zhang, S.; Chen, X.; Yang, Y. Additive-modulated switchable reaction pathway in the addition of alkynes with organosilanes catalyzed by supported Pd nanoparticles: hydrosilylation versus semihydrogenation. *Catal. Sci. Technol.* **2018**, *8*, 1039-1050.

Capsule structure

```
# parm                                        (atomic charges)
ATOM      1 C1   PAL     1      -5.720  -4.039  -4.047  0.2166
ATOM      2 C2   PAL     1      -6.117  -5.379  -4.209 -0.1611
ATOM      3 H2   PAL     1      -5.728  -6.154  -3.566  0.1472
ATOM      4 C3   PAL     1      -7.011  -5.709  -5.160 -0.1987
ATOM      5 H3   PAL     1      -7.307  -6.744  -5.280  0.1583
ATOM      6 C4   PAL     1      -7.553  -4.766  -5.955 -0.0923
ATOM      7 H4   PAL     1      -8.274  -5.048  -6.711  0.1411
ATOM      8 C5   PAL     1      -7.203  -3.416  -5.798 -0.1987
ATOM      9 H5   PAL     1      -7.658  -2.662  -6.428  0.1583
ATOM     10 C6   PAL     1      -6.311  -3.058  -4.859 -0.1611
ATOM     11 H6   PAL     1      -6.058  -2.013  -4.729  0.1472
ATOM     12 C7   PAL     1      -3.044  -2.721  -3.397  0.2166
ATOM     13 C8   PAL     1      -2.922  -2.436  -4.770 -0.1611
ATOM     14 H8   PAL     1      -3.727  -2.662  -5.453  0.1472
ATOM     15 C9   PAL     1      -1.792  -1.890  -5.264 -0.1987
ATOM     16 H9   PAL     1      -1.724  -1.678  -6.324  0.1583
ATOM     17 C10  PAL     1      -0.743  -1.610  -4.467 -0.0923
ATOM     18 H10  PAL     1       0.153  -1.174  -4.889  0.1411
ATOM     19 C11  PAL     1      -0.798  -1.887  -3.096 -0.1987
ATOM     20 H11  PAL     1       0.053  -1.672  -2.461  0.1583
ATOM     21 C12  PAL     1      -1.955  -2.452  -2.556  0.2707
ATOM     22 H12  PAL     1      -1.995  -2.689  -1.504  0.1472
ATOM     23 C13  PAL     1      -8.795  -0.012  -1.354  0.2166
ATOM     24 C14  PAL     1      -8.761  -0.558  -2.644 -0.1611
ATOM     25 H14  PAL     1      -7.909  -1.140  -2.970  0.1472
ATOM     26 C15  PAL     1      -9.817  -0.329  -3.535 -0.1987
ATOM     27 H15  PAL     1      -9.782  -0.761  -4.527  0.1583
ATOM     28 C16  PAL     1     -10.866   0.429  -3.173 -0.0923
ATOM     29 H16  PAL     1     -11.673   0.601  -3.874  0.1411
ATOM     30 C17  PAL     1     -10.918   1.012  -1.900 -0.1987
ATOM     31 H17  PAL     1     -11.759   1.636  -1.625  0.1583
ATOM     32 C18  PAL     1      -9.878   0.799  -0.990 -0.1611
ATOM     33 H18  PAL     1      -9.915   1.282  -0.026  0.1472
ATOM     34 C19  PAL     1      -8.022  -0.979   1.372  0.2166
ATOM     35 C20  PAL     1      -7.682  -2.270   1.789 -0.1611
ATOM     36 H20  PAL     1      -7.056  -2.897   1.183  0.1472
ATOM     37 C21  PAL     1      -8.151  -2.751   3.019 -0.1987
ATOM     38 H21  PAL     1      -7.889  -3.753   3.335  0.1583
ATOM     39 C22  PAL     1      -8.915  -1.976   3.812 -0.0923
ATOM     40 H22  PAL     1      -9.263  -2.366   4.761  0.1411
ATOM     41 C23  PAL     1      -9.243  -0.721   3.451 -0.1987
ATOM     42 H23  PAL     1      -9.844  -0.110   4.113  0.1583
ATOM     43 C24  PAL     1      -8.803  -0.191   2.231 -0.1611
ATOM     44 H24  PAL     1      -9.037   0.835   1.987  0.1472
ATOM     45 P1   PAL     1      -4.562  -3.520  -2.705 -0.0450
ATOM     46 P2   PAL     1      -7.370  -0.282  -0.214 -0.0450
ATOM     47 Cl1  PAL     1      -4.163  -0.315  -1.434 -0.4927
```

ATOM	48	Cl2	PAL	1	-7.671	-3.489	-1.402	-0.4927
ATOM	49	Pd	PAL	1	-5.912	-1.902	-1.400	0.4534
ATOM	50	C1	CPM	2	-4.045	-4.960	-1.658	0.3248
ATOM	51	C2	CPM	2	-4.265	-4.948	-0.269	-0.0432
ATOM	52	C3	CPM	2	-3.790	-6.011	0.539	-0.0642
ATOM	53	C4	CPM	2	-3.105	-7.026	-0.031	-0.2758
ATOM	54	H4	CPM	2	-2.686	-7.797	0.592	0.1540
ATOM	55	C3	CPM	2	-2.925	-7.109	-1.423	-0.0642
ATOM	56	C2	CPM	2	-3.342	-6.032	-2.235	-0.0432
ATOM	57	C7	CPM	2	-1.745	-5.786	-3.989	0.1226
ATOM	58	HA	CPM	2	-1.747	-5.125	-4.880	0.0963
ATOM	59	HB	CPM	2	-1.196	-5.221	-3.195	0.0963
ATOM	60	C8	CPM	2	-0.232	-7.478	-3.342	0.2127
ATOM	61	C8	CPM	2	-0.757	-8.044	-2.160	-0.1355
ATOM	62	C10	CPM	2	0.107	-8.290	-1.079	-0.2016
ATOM	63	HC	CPM	2	-0.297	-8.611	-0.132	0.1540
ATOM	64	C11	CPM	2	1.490	-8.098	-1.219	-0.1145
ATOM	65	C12	CPM	2	1.974	-7.573	-2.433	0.2707
ATOM	66	C13	CPM	2	1.090	-7.282	-3.477	-0.2804
ATOM	67	HD	CPM	2	1.471	-6.819	-4.379	0.1776
ATOM	68	C14	CPM	2	3.572	-5.924	-1.927	0.3034
ATOM	69	HF	CPM	2	2.652	-5.295	-1.831	0.0663
ATOM	70	HG	CPM	2	4.265	-5.359	-2.582	0.0663
ATOM	71	C15	CPM	2	3.357	-6.014	0.446	0.0829
ATOM	72	C16	CPM	2	2.491	-7.081	0.799	-0.1145
ATOM	73	C17	CPM	2	1.620	-6.916	1.894	-0.2601
ATOM	74	HH	CPM	2	0.912	-7.692	2.131	0.1540
ATOM	75	C16	CPM	2	1.659	-5.804	2.658	-0.1145
ATOM	76	C15	CPM	2	2.506	-4.738	2.280	0.0829
ATOM	77	C20	CPM	2	3.368	-4.877	1.181	0.2515
ATOM	78	C21	CPM	2	1.348	-2.707	2.709	0.3034
ATOM	79	HI	CPM	2	0.878	-2.957	1.724	0.0663
ATOM	80	HJ	CPM	2	1.687	-1.655	2.619	0.0663
ATOM	81	C22	CPM	2	-0.686	-3.612	3.522	0.2707
ATOM	82	C23	CPM	2	-0.547	-5.016	3.480	-0.1476
ATOM	83	C24	CPM	2	-1.619	-5.781	2.987	0.2016
ATOM	84	HK	CPM	2	-1.493	-6.841	2.833	0.1540
ATOM	85	C25	CPM	2	-2.842	-5.164	2.658	-0.1355
ATOM	86	C26	CPM	2	-2.949	-3.760	2.769	0.2127
ATOM	87	C27	CPM	2	-1.911	-3.021	3.198	-0.2804
ATOM	88	HL	CPM	2	-2.002	-1.942	3.231	0.1776
ATOM	89	C28	CPM	2	-4.155	-2.895	0.934	0.1226
ATOM	90	HM	CPM	2	-3.137	-2.838	0.483	0.0963
ATOM	91	HN	CPM	2	-4.627	-1.911	0.762	0.0963
ATOM	92	C29	CPM	2	-2.246	-8.313	-2.054	0.4000
ATOM	93	HO	CPM	2	-2.661	-8.420	-3.081	0.0200
ATOM	94	C30	CPM	2	-2.595	-9.670	-1.384	-0.3500
ATOM	95	HP	CPM	2	-2.195	-9.733	-0.353	0.1000
ATOM	96	HQ	CPM	2	-3.701	-9.759	-1.310	0.1000
ATOM	97	C31	CPM	2	-2.062	-10.863	-2.195	0.0000

ATOM	98	HR	CPM	2	-2.591	-10.907	-3.172	0.0000
ATOM	99	HS	CPM	2	-0.975	-10.732	-2.391	0.0000
ATOM	100	C32	CPM	2	-2.265	-12.181	-1.439	0.0000
ATOM	101	HT	CPM	2	-3.344	-12.315	-1.208	0.0000
ATOM	102	HU	CPM	2	-1.699	-12.142	-0.482	0.0000
ATOM	103	C33	CPM	2	-1.773	-13.371	-2.270	0.0000
ATOM	104	HV	CPM	2	-0.712	-13.214	-2.565	0.0000
ATOM	105	HW	CPM	2	-2.383	-13.458	-3.195	0.0000
ATOM	106	C34	CPM	2	-1.876	-14.674	-1.483	0.0000
ATOM	107	HX	CPM	2	-1.244	-14.624	-0.571	0.0000
ATOM	108	HY	CPM	2	-1.523	-15.518	-2.113	0.0000
ATOM	109	HZ	CPM	2	-2.930	-14.865	-1.190	0.0000
ATOM	110	C35	CPM	2	2.445	-8.336	-0.061	0.4000
ATOM	111	H1A	CPM	2	3.468	-8.489	-0.471	0.0200
ATOM	112	C36	CPM	2	2.171	-9.646	0.726	-0.3500
ATOM	113	H1B	CPM	2	2.904	-9.719	1.559	0.1000
ATOM	114	H1C	CPM	2	1.160	-9.649	1.178	0.1000
ATOM	115	C37	CPM	2	2.309	-10.894	-0.163	0.0000
ATOM	116	H1D	CPM	2	3.347	-10.943	-0.554	0.0000
ATOM	117	H1E	CPM	2	1.609	-10.820	-1.023	0.0000
ATOM	118	C38	CPM	2	1.990	-12.171	0.629	0.0000
ATOM	119	H1F	CPM	2	2.722	-12.297	1.457	0.0000
ATOM	120	H1G	CPM	2	0.980	-12.070	1.082	0.0000
ATOM	121	C39	CPM	2	1.982	-13.427	-0.256	0.0000
ATOM	122	H1H	CPM	2	1.559	-14.269	0.333	0.0000
ATOM	123	H1I	CPM	2	1.323	-13.270	-1.136	0.0000
ATOM	124	C40	CPM	2	3.379	-13.825	-0.729	0.0000
ATOM	125	H1J	CPM	2	3.787	-13.069	-1.431	0.0000
ATOM	126	H1K	CPM	2	3.324	-14.798	-1.262	0.0000
ATOM	127	H1L	CPM	2	4.066	-13.936	0.137	0.0000
ATOM	128	C41	CPM	2	0.770	-5.655	3.884	0.4000
ATOM	129	H1M	CPM	2	1.267	-4.946	4.577	0.0200
ATOM	130	C42	CPM	2	0.597	-6.937	4.746	-0.3500
ATOM	131	H1N	CPM	2	-0.033	-6.696	5.629	0.1000
ATOM	132	H1O	CPM	2	0.042	-7.719	4.191	0.1000
ATOM	133	C43	CPM	2	1.923	-7.566	5.224	0.0000
ATOM	134	H1P	CPM	2	2.500	-7.917	4.342	0.0000
ATOM	135	H1Q	CPM	2	1.688	-8.477	5.815	0.0000
ATOM	136	C44	CPM	2	2.833	-6.643	6.058	0.0000
ATOM	137	H1R	CPM	2	3.728	-7.224	6.370	0.0000
ATOM	138	H1S	CPM	2	3.210	-5.817	5.419	0.0000
ATOM	139	C45	CPM	2	2.172	-6.045	7.313	0.0000
ATOM	140	H1T	CPM	2	2.913	-5.381	7.810	0.0000
ATOM	141	H1U	CPM	2	1.305	-5.410	7.036	0.0000
ATOM	142	C46	CPM	2	1.735	-7.109	8.318	0.0000
ATOM	143	H1V	CPM	2	0.891	-7.706	7.915	0.0000
ATOM	144	H1W	CPM	2	1.392	-6.616	9.252	0.0000
ATOM	145	H1X	CPM	2	2.582	-7.783	8.564	0.0000
ATOM	146	C47	CPM	2	-3.990	-5.952	2.050	0.4000
ATOM	147	H1Y	CPM	2	-4.929	-5.390	2.246	0.0200

ATOM	148	C48	CPM	2	-4.250	-7.311	2.754	-0.3500
ATOM	149	H1Z	CPM	2	-4.382	-7.119	3.842	0.1000
ATOM	150	H2A	CPM	2	-3.390	-8.003	2.656	0.1000
ATOM	151	C49	CPM	2	-5.516	-8.003	2.225	0.0000
ATOM	152	H2B	CPM	2	-6.392	-7.334	2.375	0.0000
ATOM	153	H2C	CPM	2	-5.408	-8.200	1.136	0.0000
ATOM	154	C50	CPM	2	-5.761	-9.331	2.950	0.0000
ATOM	155	H2D	CPM	2	-5.884	-9.139	4.038	0.0000
ATOM	156	H2E	CPM	2	-4.882	-9.997	2.805	0.0000
ATOM	157	C51	CPM	2	-7.016	-10.027	2.412	0.0000
ATOM	158	H2F	CPM	2	-7.901	-9.369	2.555	0.0000
ATOM	159	H2G	CPM	2	-6.897	-10.226	1.324	0.0000
ATOM	160	C52	CPM	2	-7.263	-11.350	3.131	0.0000
ATOM	161	H2H	CPM	2	-6.402	-12.037	2.982	0.0000
ATOM	162	H2I	CPM	2	-8.176	-11.832	2.721	0.0000
ATOM	163	H2I	CPM	2	-7.411	-11.177	4.218	0.0000
ATOM	164	O1	CPM	2	-3.104	-6.044	-3.621	-0.4000
ATOM	165	O2	CPM	2	-1.090	-7.010	-4.345	-0.4000
ATOM	166	O3	CPM	2	3.311	-7.183	-2.553	-0.4000
ATOM	167	O4	CPM	2	4.212	-6.129	-0.664	-0.4000
ATOM	168	O5	CPM	2	2.492	-3.524	2.985	-0.4000
ATOM	169	O6	CPM	2	0.414	-2.786	3.794	-0.4000
ATOM	170	O7	CPM	2	-4.114	-3.109	2.348	-0.4000
ATOM	171	O7	CPM	2	-4.973	-3.885	0.306	-0.4000
ATOM	172	C1	CPM	3	-6.611	1.379	0.112	0.3248
ATOM	173	C2	CPM	3	-6.737	2.412	-0.840	-0.0432
ATOM	174	C3	CPM	3	-6.615	3.762	-0.441	-0.0642
ATOM	175	C4	CPM	3	-6.246	4.051	0.879	-0.2758
ATOM	176	H4	CPM	3	-6.099	5.076	1.176	0.1540
ATOM	177	C3	CPM	3	-6.064	3.027	1.818	-0.0642
ATOM	178	C2	CPM	3	-6.145	1.684	1.404	-0.0432
ATOM	179	C7	CPM	3	-4.342	0.625	2.549	0.1226
ATOM	180	HA	CPM	3	-3.751	1.152	1.759	0.0963
ATOM	181	HB	CPM	3	-4.002	-0.427	2.515	0.0963
ATOM	182	C8	CPM	3	-3.538	2.433	3.865	0.2127
ATOM	183	C8	CPM	3	-4.337	3.531	3.499	-0.1355
ATOM	184	C10	CPM	3	-3.725	4.770	3.263	-0.2016
ATOM	185	HC	CPM	3	-4.320	5.567	2.846	0.1540
ATOM	186	C11	CPM	3	-2.416	4.979	3.526	-0.1145
ATOM	187	C12	CPM	3	-1.612	3.905	3.991	0.2707
ATOM	188	C13	CPM	3	-2.187	2.638	4.165	-0.2804
ATOM	189	HD	CPM	3	-1.572	1.805	4.479	0.1776
ATOM	190	C14	CPM	3	0.649	4.495	3.272	0.3034
ATOM	191	HF	CPM	3	1.034	5.519	3.469	0.0663
ATOM	192	HG	CPM	3	1.536	3.831	3.311	0.0663
ATOM	193	C15	CPM	3	-0.314	5.532	1.293	0.0829
ATOM	194	C16	CPM	3	-1.384	6.341	1.751	-0.1145
ATOM	195	C17	CPM	3	-2.111	7.071	0.790	-0.2601
ATOM	196	HH	CPM	3	-3.013	7.572	1.084	0.1540
ATOM	197	C16	CPM	3	-1.724	7.095	-0.566	-0.1145

ATOM	198	C15	CPM	3	-0.595	6.447	-0.947	0.0829
ATOM	199	C20	CPM	3	0.147	5.693	-0.019	0.2515
ATOM	200	C21	CPM	3	-0.769	5.491	-3.120	0.3034
ATOM	201	HI	CPM	3	0.002	5.131	-3.831	0.0663
ATOM	202	HJ	CPM	3	-1.097	4.592	-2.541	0.0663
ATOM	203	C22	CPM	3	-3.101	5.853	-3.318	0.2707
ATOM	204	C23	CPM	3	-3.514	6.593	-2.185	-0.1476
ATOM	205	C24	CPM	3	-4.709	6.231	-1.539	0.2016
ATOM	206	HK	CPM	3	-4.980	6.708	-0.612	0.1540
ATOM	207	C25	CPM	3	-5.548	5.251	-2.092	-0.1355
ATOM	208	C26	CPM	3	-5.115	4.560	-3.242	0.2127
ATOM	209	C27	CPM	3	-3.891	4.893	-3.835	-0.2804
ATOM	210	HL	CPM	3	-3.553	4.332	-4.697	0.1776
ATOM	211	C28	CPM	3	-5.761	2.287	-3.000	0.1226
ATOM	212	HM	CPM	3	-5.683	1.434	-3.705	0.0963
ATOM	213	HN	CPM	3	-4.822	2.264	-2.393	0.0963
ATOM	214	C29	CPM	3	-5.821	3.339	3.280	0.4000
ATOM	215	HO	CPM	3	-6.137	2.460	3.885	0.0200
ATOM	216	C30	CPM	3	-6.693	4.490	3.847	-0.3500
ATOM	217	HP	CPM	3	-6.416	4.653	4.912	0.1000
ATOM	218	HQ	CPM	3	-6.499	5.442	3.320	0.1000
ATOM	219	C31	CPM	3	-8.195	4.153	3.783	0.0000
ATOM	220	HR	CPM	3	-8.484	3.825	2.761	0.0000
ATOM	221	HS	CPM	3	-8.394	3.302	4.470	0.0000
ATOM	222	C32	CPM	3	-9.078	5.338	4.207	0.0000
ATOM	223	HT	CPM	3	-10.084	4.943	4.470	0.0000
ATOM	224	HU	CPM	3	-8.657	5.820	5.116	0.0000
ATOM	225	C33	CPM	3	-9.238	6.374	3.084	0.0000
ATOM	226	HV	CPM	3	-9.671	5.885	2.184	0.0000
ATOM	227	HW	CPM	3	-8.253	6.804	2.807	0.0000
ATOM	228	C34	CPM	3	-10.158	7.513	3.515	0.0000
ATOM	229	HX	CPM	3	-11.170	7.121	3.754	0.0000
ATOM	230	HY	CPM	3	-10.250	8.252	2.691	0.0000
ATOM	231	HZ	CPM	3	-9.743	8.027	4.407	0.0000
ATOM	232	C35	CPM	3	-1.796	6.327	3.221	0.4000
ATOM	233	H1A	CPM	3	-0.883	6.442	3.831	0.0200
ATOM	234	C36	CPM	3	-2.656	7.526	3.717	-0.3500
ATOM	235	H1B	CPM	3	-2.924	7.346	4.782	0.1000
ATOM	236	H1C	CPM	3	-3.613	7.615	3.166	0.1000
ATOM	237	C37	CPM	3	-1.895	8.861	3.639	0.0000
ATOM	238	H1D	CPM	3	-1.009	8.819	4.309	0.0000
ATOM	239	H1E	CPM	3	-1.538	9.036	2.602	0.0000
ATOM	240	C38	CPM	3	-2.792	10.032	4.058	0.0000
ATOM	241	H1F	CPM	3	-3.137	9.876	5.104	0.0000
ATOM	242	H1G	CPM	3	-3.683	10.069	3.393	0.0000
ATOM	243	C39	CPM	3	-2.037	11.363	3.964	0.0000
ATOM	244	H1H	CPM	3	-1.685	11.522	2.922	0.0000
ATOM	245	H1I	CPM	3	-1.151	11.340	4.636	0.0000
ATOM	246	C40	CPM	3	-2.931	12.533	4.365	0.0000
ATOM	247	H1J	CPM	3	-3.812	12.594	3.691	0.0000

ATOM	248	H1K	CPM	3	-2.359	13.481	4.288	0.0000
ATOM	249	H1L	CPM	3	-3.278	12.412	5.414	0.0000
ATOM	250	C41	CPM	3	-2.631	7.704	-1.629	0.4000
ATOM	251	H1M	CPM	3	-1.985	8.087	-2.449	0.0200
ATOM	252	C42	CPM	3	-3.417	8.964	-1.174	-0.3500
ATOM	253	H1N	CPM	3	-4.133	8.732	-0.362	0.1000
ATOM	254	H1O	CPM	3	-2.695	9.709	-0.773	0.1000
ATOM	255	C43	CPM	3	-4.198	9.608	-2.332	0.0000
ATOM	256	H1P	CPM	3	-3.484	9.972	-3.103	0.0000
ATOM	257	H1Q	CPM	3	-4.863	8.851	-2.801	0.0000
ATOM	258	C44	CPM	3	-5.057	10.776	-1.836	0.0000
ATOM	259	H1R	CPM	3	-5.735	10.418	-1.029	0.0000
ATOM	260	H1S	CPM	3	-4.397	11.568	-1.418	0.0000
ATOM	261	C45	CPM	3	-5.901	11.360	-2.975	0.0000
ATOM	262	H1T	CPM	3	-6.549	10.565	-3.407	0.0000
ATOM	263	H1U	CPM	3	-5.234	11.746	-3.775	0.0000
ATOM	264	C46	CPM	3	-6.788	12.496	-2.475	0.0000
ATOM	265	H1V	CPM	3	-7.488	12.124	-1.697	0.0000
ATOM	266	H1W	CPM	3	-7.382	12.903	-3.321	0.0000
ATOM	267	H1X	CPM	3	-6.167	13.313	-2.052	0.0000
ATOM	268	C47	CPM	3	-6.866	4.876	-1.443	0.4000
ATOM	269	H1Y	CPM	3	-7.538	4.475	-2.234	0.0200
ATOM	270	C48	CPM	3	-7.672	6.073	-0.867	-0.3500
ATOM	271	H1Z	CPM	3	-8.600	5.670	-0.408	0.1000
ATOM	272	H2A	CPM	3	-7.116	6.599	-0.066	0.1000
ATOM	273	C49	CPM	3	-8.061	7.088	-1.960	0.0000
ATOM	274	H2B	CPM	3	-8.788	6.628	-2.662	0.0000
ATOM	275	H2C	CPM	3	-7.163	7.354	-2.557	0.0000
ATOM	276	C50	CPM	3	-8.624	8.400	-1.386	0.0000
ATOM	277	H2D	CPM	3	-7.853	8.868	-0.736	0.0000
ATOM	278	H2E	CPM	3	-8.809	9.105	-2.225	0.0000
ATOM	279	C51	CPM	3	-9.919	8.240	-0.573	0.0000
ATOM	280	H2F	CPM	3	-10.200	9.237	-0.169	0.0000
ATOM	281	H2G	CPM	3	-9.752	7.573	0.297	0.0000
ATOM	282	C52	CPM	3	-11.087	7.714	-1.406	0.0000
ATOM	283	H2H	CPM	3	-11.220	8.330	-2.321	0.0000
ATOM	284	H2I	CPM	3	-12.020	7.764	-0.806	0.0000
ATOM	285	H2I	CPM	3	-10.919	6.655	-1.692	0.0000
ATOM	286	O1	CPM	3	-5.754	0.652	2.278	-0.4000
ATOM	287	O2	CPM	3	-4.064	1.131	3.865	-0.4000
ATOM	288	O3	CPM	3	-0.251	4.072	4.315	-0.4000
ATOM	289	O4	CPM	3	0.082	4.361	1.967	-0.4000
ATOM	290	O5	CPM	3	-0.170	6.487	-2.286	-0.4000
ATOM	291	O6	CPM	3	-1.837	6.064	-3.885	-0.4000
ATOM	292	O7	CPM	3	-5.867	3.495	-3.765	-0.4000
ATOM	293	O7	CPM	3	-6.934	2.098	-2.194	-0.4000
ATOM	294	C1	CAL	4	5.520	-3.878	1.055	0.0798
ATOM	295	HA	CAL	4	5.945	-4.790	0.584	0.0800
ATOM	296	HB	CAL	4	5.671	-3.968	2.152	0.0800
ATOM	297	O9	CAL	4	4.124	-3.768	0.777	-0.3587

ATOM	298	O10	CAL	4	1.352	5.086	-0.394	-0.3587
ATOM	299	C2	CAL	4	2.426	6.031	-0.435	0.0798
ATOM	300	C3	CAL	4	3.733	5.346	-0.753	-0.1275
ATOM	301	HC	CAL	4	2.486	6.571	0.536	0.0800
ATOM	302	HD	CAL	4	2.237	6.795	-1.221	0.0800
ATOM	303	C4	CAL	4	3.903	4.721	-2.001	0.2285
ATOM	304	C5	CAL	4	4.805	5.363	0.172	0.2285
ATOM	305	C6	CAL	4	5.167	4.212	-2.361	-0.1800
ATOM	306	O4	CAL	4	2.801	4.554	-2.856	-0.4100
ATOM	307	C7	CAL	4	6.021	4.857	-0.161	-0.1800
ATOM	308	O5	CAL	4	4.594	5.865	1.467	-0.4100
ATOM	309	C8	CAL	4	6.198	4.237	-1.412	-0.1600
ATOM	310	C9	CAL	4	5.381	3.614	-3.741	0.4000
ATOM	311	C10	CAL	4	2.309	3.208	-2.891	0.4771
ATOM	312	C11	CAL	4	7.156	4.882	0.854	0.4000
ATOM	313	C12	CAL	4	4.354	4.844	2.443	0.4771
ATOM	314	HE	CAL	4	7.135	3.758	-1.642	0.0000
ATOM	315	C13	CAL	4	5.100	2.120	-3.687	-0.1800
ATOM	316	HF	CAL	4	4.635	4.065	-4.431	0.0200
ATOM	317	C14	CAL	4	6.737	3.980	-4.404	-0.3500
ATOM	318	HG	CAL	4	1.201	3.236	-2.875	0.0250
ATOM	319	HH	CAL	4	2.599	2.627	-1.979	0.0250
ATOM	320	O3	CAL	4	2.724	2.563	-4.101	-0.4100
ATOM	321	C15	CAL	4	7.104	3.598	1.665	-0.1800
ATOM	322	HI	CAL	4	6.985	5.732	1.551	0.0200
ATOM	323	C16	CAL	4	8.543	5.214	0.238	-0.3500
ATOM	324	HJ	CAL	4	3.552	5.203	3.118	0.0250
ATOM	325	H	CAL	4	3.972	3.896	1.989	0.0250
ATOM	326	O6	CAL	4	5.528	4.628	3.234	-0.4100
ATOM	327	C17	CAL	4	6.068	1.234	-3.356	-0.1600
ATOM	328	C18	CAL	4	3.781	1.660	-3.918	0.3230
ATOM	329	HK	CAL	4	6.790	3.479	-5.396	0.1000
ATOM	330	HL	CAL	4	7.599	3.618	-3.810	0.1000
ATOM	331	C19	CAL	4	6.885	5.496	-4.613	0.0000
ATOM	332	C20	CAL	4	6.298	3.527	2.826	0.3230
ATOM	333	C21	CAL	4	7.806	2.454	1.252	-0.1600
ATOM	334	HM	CAL	4	8.868	4.444	-0.488	0.1000
ATOM	335	HN	CAL	4	8.461	6.170	-0.324	0.1000
ATOM	336	C22	CAL	4	9.632	5.373	1.314	0.0000
ATOM	337	C23	CAL	4	5.822	-0.155	-3.322	-0.1800
ATOM	338	HO	CAL	4	7.049	1.596	-3.098	0.1600
ATOM	339	C24	CAL	4	3.502	0.293	-3.884	-0.3200
ATOM	340	HP	CAL	4	6.061	5.862	-5.263	0.0000
ATOM	341	HQ	CAL	4	6.818	6.017	-3.633	0.0000
ATOM	342	C25	CAL	4	8.232	5.832	-5.264	0.0000
ATOM	343	C26	CAL	4	6.255	2.398	3.559	-0.3200
ATOM	344	HR	CAL	4	8.357	2.468	0.326	0.1600
ATOM	345	C27	CAL	4	7.807	1.296	2.042	-0.1800
ATOM	346	HS	CAL	4	9.400	6.259	1.944	0.0000
ATOM	347	HT	CAL	4	9.650	4.476	1.968	0.0000

```
ATOM    348 C28  CAL     4      11.016   5.542   0.678  0.0000
ATOM    349 C29  CAL     4       4.510  -0.618  -3.564  0.3230
ATOM    350 C30  CAL     4       6.919  -1.149  -2.973  0.4000
ATOM    351 HU   CAL     4       2.489  -0.056  -4.040  0.1818
ATOM    352 HV   CAL     4       8.299   5.329  -6.254  0.0000
ATOM    353 HW   CAL     4       9.056   5.455  -4.618  0.0000
ATOM    354 C31  CAL     4       8.390   7.345  -5.450  0.0000
ATOM    355 C32  CAL     4       6.976   1.256   3.181  0.3230
ATOM    356 HX   CAL     4       5.626   2.357   4.439  0.1818
ATOM    357 C33  CAL     4       8.628   0.081   1.656  0.4000
ATOM    358 HY   CAL     4      11.227   4.669   0.022  0.0000
ATOM    359 HZ   CAL     4      11.026   6.463   0.054  0.0000
ATOM    360 C34  CAL     4      12.105   5.640   1.752  0.0000
ATOM    361 O2   CAL     4       4.178  -1.970  -3.409 -0.4100
ATOM    362 C35  CAL     4       6.944  -1.375  -1.466 -0.1800
ATOM    363 H1A  CAL     4       6.657  -2.113  -3.463  0.0200
ATOM    364 C36  CAL     4       8.300  -0.801  -3.591 -0.3500
ATOM    365 H1B  CAL     4       8.324   7.854  -4.463  0.0000
ATOM    366 H1C  CAL     4       7.575   7.730  -6.100  0.0000
ATOM    367 C37  CAL     4       9.734   7.684  -6.088  0.0000
ATOM    368 O7   CAL     4       6.837   0.078   3.934 -0.4100
ATOM    369 C38  CAL     4       7.785  -0.800   0.754 -0.1800
ATOM    370 H1D  CAL     4       8.851  -0.489   2.584  0.0200
ATOM    371 C39  CAL     4      10.035   0.390   1.076 -0.3500
ATOM    372 H1E  CAL     4      12.064   4.747   2.412  0.0000
ATOM    373 H1F  CAL     4      11.936   6.545   2.374  0.0000
ATOM    374 C40  CAL     4      13.493   5.719   1.123  0.0000
ATOM    375 C41  CAL     4       4.106  -2.377  -2.039  0.4771
ATOM    376 C42  CAL     4       7.705  -0.551  -0.624 -0.1600
ATOM    377 C43  CAL     4       6.181  -2.417  -0.898  0.2285
ATOM    378 H1G  CAL     4       8.170  -0.638  -4.684  0.1000
ATOM    379 H1H  CAL     4       8.699   0.142  -3.178  0.1000
ATOM    380 C44  CAL     4       9.327  -1.931  -3.390  0.0000
ATOM    381 H1I  CAL     4      10.567   7.332  -5.444  0.0000
ATOM    382 H1J  CAL     4       9.821   8.785  -6.210  0.0000
ATOM    383 H1K  CAL     4       9.817   7.208  -7.088  0.0000
ATOM    384 C45  CAL     4       6.045  -0.931   3.293  0.4771
ATOM    385 C46  CAL     4       7.000  -1.828   1.317  0.2285
ATOM    386 H1L  CAL     4      10.516  -0.579   0.821  0.1000
ATOM    387 H1M  CAL     4       9.979   0.979   0.140  0.1000
ATOM    388 C47  CAL     4      10.929   1.134   2.086  0.0000
ATOM    389 H1N  CAL     4      13.699   4.805   0.527  0.0000
ATOM    390 H1O  CAL     4      14.259   5.801   1.923  0.0000
ATOM    391 H1P  CAL     4      13.568   6.612   0.466  0.0000
ATOM    392 H1Q  CAL     4       3.937  -1.514  -1.347  0.0250
ATOM    393 H1R  CAL     4       3.214  -3.021  -1.915  0.0250
ATOM    394 O1   CAL     4       5.275  -3.133  -1.695 -0.4100
ATOM    395 H1S  CAL     4       8.224   0.297  -1.039  0.0000
ATOM    396 C48  CAL     4       6.249  -2.683   0.485 -0.1275
ATOM    397 H1T  CAL     4       9.330  -2.284  -2.336  0.0000
```

```
ATOM    398 H1U  CAL     4       9.027  -2.795  -4.023  0.0000
ATOM    399 C49  CAL     4      10.750  -1.504  -3.785  0.0000
ATOM    400 H1V  CAL     4       5.412  -1.418   4.064  0.0250
ATOM    401 H1W  CAL     4       5.330  -0.495   2.551  0.0250
ATOM    402 O8   CAL     4       6.892  -1.934   2.713 -0.4100
ATOM    403 H1X  CAL     4      11.135   0.485   2.963  0.0000
ATOM    404 H1Y  CAL     4      10.391   2.028   2.466  0.0000
ATOM    405 C50  CAL     4      12.248   1.630   1.464  0.0000
ATOM    406 H1Z  CAL     4      11.355  -2.423  -3.948  0.0000
ATOM    407 H2A  CAL     4      10.725  -0.942  -4.745  0.0000
ATOM    408 C51  CAL     4      11.427  -0.660  -2.693  0.0000
ATOM    409 H2B  CAL     4      12.013   2.308   0.615  0.0000
ATOM    410 H2C  CAL     4      12.791   2.237   2.220  0.0000
ATOM    411 C52  CAL     4      13.180   0.515   0.958  0.0000
ATOM    412 H2D  CAL     4      11.449  -1.230  -1.739  0.0000
ATOM    413 H2F  CAL     4      10.864   0.281  -2.522  0.0000
ATOM    414 C53  CAL     4      12.857  -0.300  -3.084  0.0000
ATOM    415 H2G  CAL     4      14.065   0.994   0.487  0.0000
ATOM    416 H2H  CAL     4      12.680  -0.087   0.172  0.0000
ATOM    417 C54  CAL     4      13.671  -0.404   2.075  0.0000
ATOM    418 H2I  CAL     4      13.466  -1.221  -3.211  0.0000
ATOM    419 H2J  CAL     4      13.317   0.321  -2.287  0.0000
ATOM    420 H2K  CAL     4      12.864   0.279  -4.032  0.0000
ATOM    421 H2L  CAL     4      14.107   0.191   2.906  0.0000
ATOM    422 H2M  CAL     4      14.455  -1.082   1.676  0.0000
ATOM    423 H2N  CAL     4      12.840  -1.030   2.461  0.0000
END
```
